# Patient- and Caregiver-Reported Experiences of Rare Diseases in Türkiye: A Patient-Organization-Recruited Online Survey

**DOI:** 10.3390/jcm15176690

**Published:** 2026-08-28

**Authors:** Murat Gülşen, Hikmet Can Çubukçu, Onur Burak Dursun, Hayri Canbaz, Sıddika Songül Yalçın

**Affiliations:** 1 Autism, Special Mental Needs and Rare Diseases Department, Turkish Ministry of Health, 06800 Ankara, Türkiye; 2 Department of Social Pediatrics, Institute of Child Health, Hacettepe University, 06230 Ankara, Türkiye; 3 Department of Medical Biochemistry, Sincan Training and Research Hospital, 06949 Ankara, Türkiye; 4 Department of Child and Adolescent Psychiatry, Faculty of Medicine, Trabzon University, 61335 Trabzon, Türkiye; 5 Department of Emergency Medicine, Yenimahalle Training and Research Hospital, University of Yıldırım Beyazıt, 06370 Ankara, Türkiye

**Keywords:** rare diseases, patient experience, health services, health care delivery, online survey

## Abstract

**Background and Objectives**: Rare diseases collectively affect large populations and require coordinated, patient- and caregiver-informed health and social support. This study had an overarching descriptive objective to characterize patient- and caregiver-reported experiences of living with rare diseases in Türkiye and a secondary exploratory objective to assess differences across broad disease categories. **Materials and Methods**: This cross-sectional, anonymous, voluntary non-probability online survey recruited affected individuals and relatives/caregivers through patient-organization networks. The 44-item questionnaire covered sociodemographic and caregiving context, diagnostic experiences, psychosocial support, healthcare access, and service-improvement priorities. Descriptive analyses, exploratory disease-group comparisons, Cramér’s V, and selected multivariable logistic regression models were used. **Results**: A total of 1642 responses were analyzed; 76.7% were completed by relatives/caregivers, and 66.4% concerned patients younger than 18 years. Among participants diagnosed after symptom onset (n = 1388), 34.4% reported an incorrect or alternative diagnosis and 64.0% sought at least one additional specialist opinion. Psychosocial support was needed by 74.2%, whereas 34.5% reported receiving professional support. Access to current treatment services was perceived as moderately adequate by 29.3% and adequate by 13.0%. The leading service-improvement priorities were increasing healthcare professionals’ knowledge (82.9%) and establishing specialized rare-disease centers (79.5%). **Conclusions**: This patient-organization-recruited survey provides a large descriptive account of the experiences and priorities reported by participating patients and caregivers. The findings identify perceived priorities, particularly clinician education and specialized centers, while not constituting nationally representative estimates.

## 1. Introduction

Rare diseases affect small patient populations, but collectively represent a substantial public health challenge. Definitions vary internationally: the United States defines a rare disease as one affecting fewer than 200,000 people, whereas the European Union uses a threshold of no more than 5 in 10,000 individuals [1]. Individually, each rare disease affects only a tiny number of people, but collectively they represent a major public health concern worldwide. Population-based estimates suggest that 3.5% to 5.9% of the global population, or approximately 300 million people, live with a rare disease [2]. The 2025 World Health Assembly resolution recognizing rare diseases as a global health priority further reflects the increasing policy attention given to this population [3].

In Türkiye, an estimated 5–7 million people are affected by rare diseases [4]. Consanguineous marriage, reported at approximately 24% in national analyses, contributes to the burden of autosomal recessive genetic conditions [5]. Türkiye has also recently revised its rare disease definition. Although a stricter national criterion was historically used [6], the 2023–2027 Rare Diseases Health Strategy Document and Action Plan aligned the official definition with the EU threshold of <1 in 2000 individuals [7].

Rare diseases are clinically heterogeneous, with more than 9000 recognized clinical entities and more than 8000 disease–gene relationships listed by Orphanet [8]. Around 70% begin in childhood, many are chronic, progressive, life-threatening, or disabling, and disease-specific treatment is available for fewer than 10% of patients [2,9,10]. These features create complex and long-term needs that extend beyond diagnosis and medication access to multidisciplinary care, psychosocial support, social services, and family-centered health policy. Because each disease may be individually unfamiliar to clinicians and policymakers, patient- and caregiver-reported experience data are particularly valuable for identifying cross-cutting system problems that affect many rare disease groups [11,12].

Given the multidimensional nature of rare-disease care, a literature-informed descriptive approach was used to examine several cross-cutting domains of patient and caregiver experience, including diagnostic pathways, psychosocial support, healthcare access, treatment experiences, and perceived priorities for service improvement. These domains were selected because previous national and international rare-disease surveys have identified them as recurring challenges across otherwise clinically heterogeneous conditions [11,13]. The study was not designed to test a formal theoretical model; rather, it aimed to provide a broad descriptive assessment of patient- and caregiver-reported experiences within the Turkish healthcare context.

The overarching objective of this study was to describe patient- and caregiver-reported experiences of living with rare diseases in Türkiye, with particular emphasis on diagnostic pathways, psychosocial support, healthcare access, treatment experiences, and perceived priorities for improving rare-disease services. A secondary, exploratory objective was to assess whether these reported experiences differed across broad rare-disease categories.

Such evidence is important for translating national rare disease strategies into services that respond to patients’ lived experiences rather than only to clinical classifications.

## 2. Materials and Methods

### 2.1. Study Design and Participants

This study was a descriptive cross-sectional, non-probability online survey of individuals affected by rare diseases in Türkiye. Eligible participants were individuals with a diagnosed rare disease and primary caregivers responding on behalf of an affected individual. People without a diagnosed rare disease were outside the scope of the study. Participation was voluntary and anonymous. Between March 2024 and February 2025, 1642 patients and caregivers completed the survey. The online format was selected to enable participation from geographically dispersed families and multiple disease communities.

The survey used a voluntary, non-probability online recruitment approach. Participants were recruited through rare-disease patient organizations and their communication networks. Participating organizations distributed the electronic survey link within their communities. Before accessing the questionnaire, participants viewed an information page explaining the study purpose and provided electronic informed consent. The survey recorded whether the respondent was the affected individual or a relative/caregiver. Although the survey platform did not technically restrict multiple submissions, the dataset was screened for potential duplicate responses by comparing combinations of case-level characteristics, including reported diagnosis, age, and other relevant patient characteristics. No records with patterns suggestive of duplicate reporting for the same patient were identified. The number of people reached through organization networks, link views, and incomplete survey starts was not recorded.

Rare-disease diagnoses were reported by respondents and were not independently verified against medical records. The questionnaire included a predefined list of rare diseases and an “Other” option through which respondents could specify diagnoses not included in the list. Because reported diagnoses were highly heterogeneous and many individual diagnoses were represented by small numbers, individual conditions were grouped into seven broad analytic categories to facilitate interpretable descriptive summaries and exploratory between-group comparisons: neuromuscular diseases; metabolic and storage diseases; neurogenetic and neurodegenerative diseases; congenital anomalies and syndromes; cardiovascular and pulmonary diseases; autoimmune, autoinflammatory, and rheumatologic diseases; and other or unspecified rare diseases. The other/unspecified group included responses without a specific diagnosis and low-frequency diagnoses not analyzed separately. Categorization was informed by Orphanet disease classification and related rare disease nomenclatures [8]. For multisystem syndromes, assignment followed the dominant clinical presentation. The complete diagnosis-to-category mapping is provided in List S1. Disease-group comparisons were exploratory and were not intended to test prespecified causal or etiological hypotheses.

### 2.2. Survey Instrument and Data Collection

Data were collected using a structured 44-item electronic survey administered in Turkish language through Google Forms. The survey covered sociodemographic characteristics, medical and caregiving context, diagnostic experiences, psychosocial needs, healthcare access, functional and social consequences, and perceived priorities for improvement. The survey was developed and administered in Turkish; the English-language version provided in Questionnaire S1 is a translation prepared for reporting and publication purposes and was not administered to participants. Items were developed from a targeted review of international and national rare disease patient-experience surveys and related instruments [4,12,14,15] and were subsequently refined by the study team to reflect the objectives of the study and the Turkish healthcare context.

The draft survey was piloted with 20 participants to assess item comprehensibility, clarity of wording and response options, and practical completion of the electronic survey. Feedback from the pilot was used to refine item wording and presentation before the main data collection. Completion time was approximately 20–30 min. The survey was designed as a multidomain descriptive instrument and was not intended to constitute a psychometric scale measuring a single latent construct. Consequently, internal-consistency reliability coefficients were not calculated for the survey as a whole. The instrument did not undergo formal psychometric validation before use.

The survey was hosted on Google Forms in an account controlled by the corresponding author, who was the only person able to view responses during data collection. Participants accessed it directly through the electronic survey link distributed by participating rare-disease patient organizations and their communication networks. Before the questionnaire opened, participants viewed an electronic information page describing the study purpose and procedures and had to provide electronic informed consent to continue. The survey did not collect direct personal identifiers. After completion of data collection, responses were exported from Google Forms to an Excel dataset and retained by the research team for data management and statistical analysis.

The survey collected patient age, sex, education, household income, employment status, respondent type, and caregiver education and employment. Medical items included disease category, family history, prenatal or newborn screening diagnosis, routine follow-up, and age at diagnosis. Other sections assessed prior awareness, information sources, psychosocial support needs and receipt of support, patient organization engagement, functional limitations, social and educational or occupational impact, employment and financial consequences, diagnostic pathway, number of physicians consulted, previous incorrect or alternative diagnoses, additional specialist opinions, perceived possibility of an earlier diagnosis, health service utilization, barriers to care, treatment adequacy, and priorities for improving rare disease services. Several items permitted multiple responses to capture the overlapping nature of medical, social, and administrative challenges experienced by individuals and families affected by rare diseases. For these items, each response option was treated as a separate binary variable, and percentages could therefore exceed 100%.

Diagnostic timing was represented by five-category variable: prenatal diagnosis, diagnosis through newborn screening at birth, diagnosis before symptom onset, diagnosis after symptom onset, or diagnosis by clinical examination at birth. Full-sample diagnostic-route results used all 1642 respondents. Analyses of the symptomatic diagnostic journey; including the number of physicians consulted before definitive diagnosis, previous incorrect or alternative diagnoses, additional specialist opinions, and perceived possibility of an earlier diagnosis were restricted to the 1388 participants classified as diagnosed after symptom onset.

Diagnostic timing was recorded using predefined categories indicating whether the definitive diagnosis was made prenatally, through screening at birth, before symptom onset, after symptom onset, or by clinical examination at birth. Because symptom-onset timing was retrospectively reported and could have occurred many years before survey participation, a calculated symptom-to-diagnosis interval is not used as a principal outcome in the analysis. Questions addressing the symptomatic diagnostic journey—including the number of physicians consulted before definitive diagnosis, previous incorrect or alternative diagnoses, additional specialist opinions, and perceived possibility of an earlier diagnosis—were analyzed only among participants whose definitive diagnosis occurred after symptom onset.

### 2.3. Data Handling, Privacy Protections and Ethical Considerations

The study was approved by the Ethics Committee of Ankara City Hospital (E2-23-5889, 27 December 2023). Electronic informed consent was obtained from all respondents. The exported dataset was stored on a password-protected, antivirus-protected personal computer under the control of both corresponding authors and was not accessible to other individuals. Because the survey did not collect direct personal identifiers, the analytic file contained no names, email addresses, or comparable directly identifying fields. Data were analyzed in aggregate and handled in accordance with applicable Turkish data-protection requirements. For multiple-response items, each response option was treated as an independent binary variable.

### 2.4. Statistics

Statistical analyses were performed using IBM SPSS Statistics for Windows, version 25 (IBM Corp., Armonk, NY, USA). Categorical variables were summarized as frequencies and percentages. Disease-group comparisons used chi-square tests; significant associations were followed by adjusted-residual post hoc analyses with Bonferroni correction. Continuous variables were assessed using histograms, Kolmogorov–Smirnov tests, and skewness and kurtosis values. Approximately normally distributed variables were reported as mean ± standard deviation, and non-normally distributed variables as medians and interquartile ranges.

Categorical variables were compared using Pearson’s chi-square test. Expected cell counts were examined for all contingency tables; when chi-square assumptions were not adequately satisfied because of sparse cells, Monte Carlo estimates were used. Effect sizes for principal categorical comparisons were quantified using Cramér’s V. Disease-group comparisons were considered exploratory, and no comprehensive adjustment for multiple testing was applied. Accordingly, individual *p* values were interpreted cautiously, and emphasis was placed on the magnitude and consistency of observed differences rather than statistical significance alone.

Disease-group comparisons were considered exploratory. Categorical variables were compared using Pearson’s chi-square test, with Monte Carlo estimates used for sparse contingency tables when expected-cell assumptions were not satisfied. Effect sizes for categorical comparisons were quantified using Cramér’s V. Given the substantial demographic and respondent-profile differences across disease categories, a limited set of clinically relevant outcomes was selected for adjusted analysis rather than fitting multivariable models for all questionnaire items. Binary outcomes were analyzed using multivariable logistic regression, and perceived adequacy of access to current treatment services was analyzed using ordinal logistic regression (Table S1). Models of diagnostic-experience outcomes were restricted to participants diagnosed after symptom onset and adjusted for age at diagnosis, sex, respondent type, and disease category. Models of current psychosocial, social, and treatment-access outcomes were adjusted for current patient age, sex, respondent type, and disease category. As a sensitivity analysis, the selected models were repeated after exclusion of the heterogeneous Other/unspecified disease category. Adjusted associations are reported as odds ratios (aORs) with 95% confidence intervals.

Analyses of the symptomatic diagnostic pathway were restricted to participants diagnosed after symptom onset (n = 1388). Multivariable models for incorrect/alternative diagnosis and perceived possibility of an earlier diagnosis included age at diagnosis, sex, respondent type, and disease category as covariates.

Statistical significance was set at *p* < 0.05. The STROBE guideline was used in this manuscript [16,17].

## 3. Results

The results are organized around five descriptive domains: sample and caregiving characteristics; disease awareness, information, psychosocial support, and patient-organization engagement; functional and social consequences; the diagnostic pathway; and healthcare utilization, access, and service-improvement priorities. Overall-sample estimates are presented as descriptions of participating respondents, while disease-group comparisons are exploratory and interpreted in light of case-mix differences.

### 3.1. Sample Characteristics and Sociodemographic Profile

Among 1642 respondents, the largest disease groups were neuromuscular diseases (n = 641; 39.0%) and metabolic and storage diseases (n = 490; 29.8%), followed by neurogenetic/neurodegenerative diseases (n = 170; 10.4%), congenital anomalies/syndromes (n = 158; 9.6%), cardiovascular/pulmonary diseases (n = 74; 4.5%), autoimmune/autoinflammatory/rheumatologic diseases (n = 64; 3.9%), and other/unspecified diseases (n = 45; 2.7%) (Table 1A).

Overall, 23.3% of surveys were completed by patients and 76.7% by relatives or caregivers. Self-response varied significantly by disease category (*p* < 0.001), from 6.3% in congenital anomalies/syndromes to 73.4% in autoimmune/autoinflammatory/rheumatologic diseases. Two-thirds of patients were children (<18 years: 66.4%), and the mean age was 15.9 ± 13.9 years. Pediatric representation was highest in congenital anomalies/syndromes and lowest in other/unspecified diseases, while autoimmune/autoinflammatory/rheumatologic diseases were predominantly adult (*p* < 0.001).

Patients were 57.9% male and 42.1% female, with significant variation by disease category (*p* < 0.001). Male predominance was most pronounced in neuromuscular diseases, whereas female proportions were higher in autoimmune/autoinflammatory/rheumatologic diseases. Patient education also differed across groups: 26.3% had no formal education, 41.7% had primary school or less, 16.1% had high school education, and 15.8% had university education (*p* < 0.001). Monthly household income was most commonly 17,000–35,000 (40.0%), followed by <17,000 (27.4%), 35,000–50,000 (19.0%), and >50,000 (13.6%) (*p* < 0.001).

### 3.2. Caregiving Context, Education, and Employment

Caregiver education was primary school or less in 30.9%, high school in 24.3%, and university or higher in 29.7%; 15.2% reported no caregiver (*p* < 0.001, Table 1B). Regarding patient working status, 43.7% were not of working age, 19.9% were students, 16.9% were unable to work, 9.3% were employed, and 2.9% were retired (*p* < 0.001). Among caregivers, 48.8% were housewives, 13.9% were employed, 4.6% were retired, and 1.6% were not working specifically to care for the child. Caregiver employment status also differed significantly by disease category (*p* < 0.001).

### 3.3. Family History, Disease Awareness, and Information Sources

Most respondents (74.7%) reported a single affected case in the family, while 25.3% reported two or more affected family members. Familial occurrence varied by diagnostic category (*p* < 0.001). Congenital anomalies/syndromes were most often single cases, whereas autoimmune/autoinflammatory/rheumatologic diseases had the highest proportion of multiple affected family members (Table 2).

Prior disease awareness was limited: 87.1% had first heard of the disease at diagnosis, with rates above 80% in all groups, and differences between disease groups were statistically significant (*p* = 0.004).

Internet and social media were the most frequent information sources (81.3%), followed by other patients or relatives (63.1%), patient organizations (53.5%), and health professionals (51.3%). Use of health professionals and patient organizations varied significantly across disease categories (*p* < 0.001) with the highest utilization reported in neuromuscular diseases (65.8%) and cardiovascular and/or pulmonary diseases (66.2%).

Routine follow-up at a health institution was reported by 82.3%, but absence of routine follow-up was more frequent in neuromuscular and neurogenetic/neurodegenerative diseases.

### 3.4. Psychosocial Needs, Support Use, and Patient Organization Engagement

Overall, 74.2% reported needing emotional, psychosocial, or psychiatric support at or after diagnosis, but only 34.5% had received support from health professionals. Need was highest in congenital anomalies/syndromes (85.4%), whereas receipt of support was highest in congenital anomalies/syndromes (48.7%) and autoimmune/autoinflammatory/rheumatologic diseases (46.9%). Lower utilization was reported in metabolic, storage and neuromuscular diseases (Table 2).

Patient association or non-governmental organization (NGO) membership/contact was reported by 50.4%, while 40.5% reported no membership and 9.1% did not know or could not find an organization. Engagement was highest in neuromuscular and cardiovascular/pulmonary diseases. Lack of awareness of relevant organizations was particularly notable in neurogenetic/neurodegenerative, autoimmune/autoinflammatory/rheumatologic, and other/unspecified groups.

### 3.5. Disease Burden, Challenges, and Diagnostic Pathway

Disease-related difficulties were common. Respondents reported limitations in social life or relationships (54.5%), academic or occupational functioning (50.9%), daily activities (49.7%), self-care (49.3%), and mobility or sensory function (37.8%); only 8.2% reported no major difficulty. After diagnosis, the most frequent consequences were restricted social life (64.8%), weakened or lost contact with relatives or friends (52.1%), financial deterioration (37.9%), reduced employment opportunities (18.1%), resignation from employment (12.2%), and relocation for hospital access or related reasons (15.7%).

The most commonly endorsed ongoing challenges were uncertainty about what to do (63.5%), difficulties during the diagnostic process (56.3%), financial difficulties (55.4%), difficulties obtaining or renewing disability or needs assessment reports (46.7%), medication access problems (44.2%), healthcare access barriers (41.0%), and frequent need for medical testing (40.6%) (Table 3C).

Of the 1642 participants, 1388 (84.5%) were diagnosed after symptom onset, 150 (9.1%) through screening at birth, 63 (3.8%) by clinical examination at birth, 31 (1.9%) before symptom onset, and 10 (0.6%) prenatally (Table 4A). The retrospectively calculated overall symptom-to-diagnosis interval is reported only as a secondary descriptive statistic: the median was 0.42 years (IQR 0.00–2.00), with a right-skewed distribution and a mean of 2.44 ± 5.69 years.

Analyses of the symptomatic diagnostic journey were restricted to the 1388 participants diagnosed after symptom onset. Among them, 133 (9.6%) consulted one physician before definitive diagnosis, 606 (43.7%) consulted 2–3 physicians, 446 (32.1%) consulted 4–10 physicians, 120 (8.6%) consulted 11–20 physicians, and 83 (6.0%) consulted more than 20 physicians. An incorrect or alternative diagnosis was reported by 478 (34.4%) participants, 1071 (77.2%) considered that the definitive diagnosis could have been made earlier, and 888 (64.0%) sought at least one additional specialist opinion (Table 4B).

In the full sample (n = 1642), perceived reasons for delayed diagnosis included lack of knowledge among healthcare professionals (49.9%), unavailability of diagnostic testing (41.0%), delays in diagnostic testing (29.3%), difficulty accessing healthcare facilities (8.8%) (Table 4C).

### 3.6. Health Service Utilization, Access to Care, and Priorities for Improvement

Perceived deficiencies in information and communication were frequent: insufficient disease information (55.5%), limited time with healthcare professionals (47.5%), lack of support or counseling services (47.3%), and communication manner (28.7%). Only 17.5% reported no deficiencies (Table 5A).

Health service utilization reflected the multisystem nature of rare diseases. Across all groups, the most frequently used services were family medicine (66.1%), emergency medicine (51.7%), neurology (50.0%), cardiology (45.1%), ophthalmology (39.5%), and physical medicine and rehabilitation (33.4%) (Table S2). Utilization patterns differed by disease category: metabolism specialists, family medicine, and emergency medicine were frequently used in metabolic and storage diseases; neurology and family medicine dominated neuromuscular and neurogenetic/neurodegenerative diseases; family medicine, cardiology, emergency medicine, and ophthalmology were common in congenital anomalies/syndromes; rheumatology was visited by the vast majority of patients with autoimmune/autoinflammatory/rheumatologic diseases; and cardiology, family medicine, emergency medicine, and pulmonology were prominent in cardiovascular/pulmonary diseases.

Perceived adequacy of access to current treatment services was rated as not adequate by 30.7%, inadequate by 27.0%, moderately adequate by 29.3%, and adequate by 13.0% (n = 1642, *p* < 0.001) (Table 5B). In the full sample (n = 1642), reported barriers included long distance to hospital (44.1%), needed services not being provided (34.7%), lack of transportation (16.9%), inability to take leave from work (16.2%), uncertainty about where or which department to apply (10.6%), and caregiving responsibilities for another person at home (9.3%).

The components considered most important during diagnosis and treatment were access to accurate information (77.1%), appropriate specialists and health professionals (75.6%), awareness of new treatment trials (73.1%), and medications (67.7%), communication with other patients/patient groups (50.4%), access to medical devices (41.1%), and home healthcare services (31.5%) (Table 5C).

Key elements to improve diagnostic and treatment service quality were most commonly identified as increasing healthcare professionals’ knowledge (82.9%), establishing specialized rare disease centers (79.5%), accelerating bureaucratic processes for access to new medications (74.4%), improving patient-provider communication (64.9%), and improving information and access to social services (61.2%), facilitating transition from pediatric to adult care (35.9%), and more effective use of family physician services (36.2%) (Table 5D).

Given marked differences in age, sex, respondent profile, and other demographic characteristics across disease categories (Table 1A,B), selected key outcomes were further examined using adjusted models. Several unadjusted disease-group differences persisted after adjustment, whereas others were attenuated, indicating that some of the crude between-group variation reflected differences in case mix. Detailed adjusted estimates are provided in Table S1. Respondent-type-stratified results for selected subjective outcomes are provided in Table S3.

## 4. Discussion

### 4.1. Demographic Profile and Respondent Characteristics

This large patient-organization-recruited survey describes patient- and caregiver-reported experiences across a broad range of rare diseases in Türkiye. Two-thirds of patients were children, consistent with the substantial pediatric burden described in international rare-disease epidemiology [13]. However, this similarity should not be interpreted as evidence that the present sample is representative of the overall rare-disease population in Türkiye. Caregivers (predominantly parents) were the respondents in 77% of cases, a pattern similarly observed in other Turkish surveys [4]. This reflects the young age of many patients and the reliance on family for care and advocacy. Respondent type varied substantially across disease categories, with relatives/caregivers more frequently responding for children and for individuals with congenital, neurogenetic/neurodegenerative, and neuromuscular conditions, whereas self-response was more common among adults and in some predominantly adult disease groups. This pattern underscores the importance of distinguishing patient self-report from caregiver proxy report when interpreting rare-disease experience data.

The sex distribution also reflected known disease patterns. Overall male predominance was driven largely by neuromuscular diseases, including X-linked conditions like Duchenne Muscular Dystrophy, while autoimmune/autoinflammatory/rheumatologic diseases were predominantly female. These patterns are clinically plausible given the heterogeneous epidemiology and age of onset of rare diseases, but they do not establish representativeness of the present sample. They also illustrate why crude disease-group comparisons require caution. Accordingly, disease-group comparisons were treated as exploratory, and selected key outcomes were additionally examined using models adjusted for relevant case-mix characteristics.

Many families reported limited education and household income, which is important because socioeconomic disadvantage can intensify rare disease burden. Financial wellbeing has been associated with lower caregiver burden and better quality of life [18]. Socioeconomic circumstances may influence families’ ability to navigate specialist care, travel, administrative processes, and long-term support needs. However, because the present study was cross-sectional, employment status and financial circumstances should be interpreted as contextual characteristics rather than as consequences directly attributable to the rare disease or caregiving.

### 4.2. Psychosocial Support Needs and Information Sources

One of the most important findings was the discrepancy between psychosocial need and support received. Nearly three-quarters of respondents reported needing emotional, psychosocial, or psychiatric support at or after diagnosis, yet only one-third had received such support from health professionals. This gap is consistent with prior studies showing that rare disease families often experience psychological distress but limited access to structured support. A recent Turkish survey found 48% of families desired psychological support but only 18–20% accessed professional help [4]. Likewise, an Australian study reported that less than half of families were offered psychological support around the time of a child’s diagnosis, despite 86% of families believing such support should always be offered [19]. The psychosocial toll of living with a rare disease is enormous: international data show that people with rare diseases experience depression and anxiety at rates several times higher than the general population [20]. This gap calls for integrating mental health services into rare disease care, as such support should not depend only on a family’s ability to request help. A European expert consensus study has urged that psychosocial support be made an integral part of rare disease management, noting that frequent misdiagnosis, uncertainty, and lack of support contribute to psychosocial vulnerability [20]. Taken together, our results and the literature point to an urgent need for structured psychosocial services (counseling, support groups, psychiatric care) for rare disease patients and caregivers, especially around the time of diagnosis when emotional distress is acute.

Information access was another major gap. More than 80% of respondents used the internet or social media, while only half identified health professionals as an information source. Online communities and patient groups can be valuable, especially when expertise or time is limited [21], but reliance on online information also increases exposure to misinformation. Our finding that 55.5% perceived insufficient disease information from professionals aligns with other countries reports that many parents are dissatisfied with providers’ knowledge or communication about rare diseases [22]. That indicates a need for better provider education, clearer communication, proper time planning and using reliable patient education materials. Information should include not only disease mechanisms, but also expected care pathways, warning signs, available social rights, rehabilitation options, school or workplace accommodations, and trustworthy sources for ongoing updates. Patient organizations already play an important role, and encouragingly, nearly half of respondents were connected to such organizations. Stronger collaboration between healthcare providers, public institutions, and patient associations could improve information quality and reduce the feeling of isolation.

### 4.3. Disease Burden, Daily Life Impact and Social Consequences

Most respondents reported at least one major difficulty attributable to the disease, most commonly limitations in social life, education or work activities, and basic daily functioning. These findings indicate that participating patients and families reported multidimensional consequences extending beyond clinical manifestations to daily functioning, social participation, education, employment, and household finances. Our findings are in line with accumulating evidence from other contexts that rare diseases cause multidimensional hardship; physically, psychologically, socially, and economically [22]. For instance, a Polish study of rare disease caregivers noted broad declines in all domains of quality of life, tied to the heavy care burden and financial stress [18].

Globally, rare disease patients frequently experience social isolation and stigma, which can lead to depression, anxiety, and reduced self-esteem [22]. In a study, 70% of Huntington’s disease patients or caregivers had to reduce or modify their employment due to disease demands, a figure echoed by our finding that many caregivers (especially mothers) are not in paid work (48.8% of caregivers in our sample identified as housewives, and some explicitly stopped working to provide care) [23]. In the present study, a substantial proportion of caregivers were not in paid employment, while some respondents explicitly reported employment consequences following diagnosis. However, employment categories such as being unemployed or a housewife cannot establish that caregiving itself caused nonemployment.

In another study, Turkish caregivers had to quit their jobs and 25% of affected children had to leave school because of their condition [4]. We similarly found a substantial minority of families reporting resignation from jobs (12%) or other explicitly reported effects on employment, education, finances, and social participation. Such disruptions compound the financial strain and can widen socioeconomic disparities.

The adjusted analysis of social-life limitation further indicated that respondent type was not independently associated with this outcome after accounting for current age, sex, and disease category. Thus, although proxy reporting remains an important interpretive consideration, the observed social-life impact was not explained solely by whether the questionnaire was completed by the patient or a relative/caregiver.

Taken together, these findings support considering rare-disease care within a broader family and social context rather than solely through clinical encounters. Respondents’ reports concerning school or work participation, financial strain, travel, administrative requirements, and social participation identify areas that warrant further longitudinal and disease-specific investigation. The reported needs for reliable information, coordinated services, emotional support, and recognition of family circumstances are interrelated dimensions of care rather than isolated service preferences.

### 4.4. Diagnostic Odyssey and Healthcare System Challenges

The diagnostic experiences reported in this survey illustrate the complexity of reaching a definitive diagnosis for many people living with rare diseases. The sample included individuals diagnosed prenatally, through newborn screening, before symptom onset, by clinical examination at birth, and after symptom onset. A retrospectively calculated overall symptom-to-diagnosis interval is presented in the results only as a secondary descriptive statistic and is not used as the principal interpretive outcome, because retrospective onset dates across a wide age range and fundamentally different diagnostic pathways are susceptible to recall error and are not directly comparable. The principal diagnostic-pathway presentation therefore distinguishes the route to diagnosis, and analyses of physician consultations, previous incorrect or alternative diagnoses, additional specialist opinions, and perceived possibility of an earlier diagnosis are restricted to the 1388 (84.5%) participants diagnosed after symptom onset.

Among participants diagnosed after symptom onset, almost half (46.7%) reported consulting four or more physicians before receiving the definitive diagnosis, including 14.6% who reported consulting more than ten. Approximately one-third (34.4%) reported having received an incorrect or alternative diagnosis before the definitive diagnosis. These findings are consistent with the broader international literature describing rare-disease diagnosis as a frequently complex pathway involving repeated consultations, referral across specialties, and previous alternative diagnoses [13,19]. However, direct comparison of diagnostic-delay durations across studies is problematic because studies differ in disease composition, definitions of symptom onset, inclusion of screening-detected conditions, and methods used to ascertain diagnostic timing.

Among those diagnosed after symptom onset, 77.2% believed that the definitive diagnosis could have been made earlier. Similar perceptions of missed opportunities or potentially earlier diagnosis have been reported internationally [19]. This measure should not, however, be interpreted as an objective demonstration of diagnostic delay; it represents the respondent’s retrospective assessment of the diagnostic experience.

Differences in perceived diagnostic delay may reflect not only objective healthcare delays but also differences in disease awareness, patient expectations, access to information through patient communities, and retrospective reinterpretation of prior clinical encounters. Structural factors such as referral pathways and specialist availability may further influence how diagnostic experiences are perceived.

The adjusted analyses also support cautious interpretation of these subjective assessments. After accounting for age at diagnosis, sex, respondent type, and disease category, respondent type was not independently associated with reporting an incorrect or alternative diagnosis. In contrast, relatives/caregivers had higher odds than patient self-respondents of reporting that the diagnosis could have been made earlier. This difference suggests that respondent perspective may influence retrospective evaluations of the diagnostic journey, particularly for more subjective outcomes.

Although international studies have also documented frequent incorrect or alternative diagnoses, differences in question wording, disease mix, recruitment, and reference populations make direct comparison of percentages inappropriate. The present finding should therefore be interpreted as evidence that previous alternative or incorrect diagnoses were commonly reported within this patient-organization-recruited sample, rather than as a population estimate for Türkiye.

In the full sample, most commonly perceived causes of delayed diagnosis were lack of physician knowledge (49.9%), difficulty accessing diagnostic tests (41.0%), and testing delays (29.3%). In Australia 69% similarly cited physicians’ lack of knowledge as a cause of delayed diagnosis [19]. This consistency with international reports suggests that respondents in different health-system contexts may perceive clinician familiarity with rare conditions and access to appropriate diagnostic pathways as important components of the diagnostic experience. Nevertheless, these responses represent respondent-attributed explanations and cannot establish that these factors objectively caused diagnostic delay.

Perceived difficulty accessing diagnostic testing also requires cautious interpretation. Newborn-screening programs differ substantially across countries in the number and type of disorders included [24], and screening can alter the diagnostic pathway by identifying selected conditions before symptoms develop. Among all participants in this study, 9.1% were diagnosed through newborn screening and a further 1.9% before symptom onset. These pathways illustrate why screening-detected and symptom-initiated diagnoses should not be combined when evaluating diagnostic experiences. Respondents’ reports of difficulty accessing diagnostic tests should likewise be interpreted as perceived barriers rather than direct evidence that specific diagnostic technologies were unavailable.

Following the initial diagnosis, 64.0% of participants in the symptom-initiated group (n = 1388) sought an additional opinion from at least one other physician. Seeking another opinion may reflect a range of motivations, including confirmation of a rare diagnosis, exploration of management options, access to disease-specific expertise, or personal preference; it should not be interpreted as evidence of dissatisfaction unless dissatisfaction was directly assessed. Consistently, “uncertainty/confusion about what to do” was the most frequently reported ongoing challenge (63.5%).

Taken together, these findings are consistent with international evidence emphasizing the importance of access to rare-disease expertise, appropriate referral pathways, diagnostic information, and continuity of guidance across the diagnostic journey [13,19,25]. In the Turkish context, respondents particularly prioritized improving healthcare professionals’ knowledge and access to specialized expertise. These findings identify respondent-reported areas for further service evaluation rather than demonstrating specific causal deficiencies in the healthcare system.

### 4.5. Healthcare Access, Treatment Gaps, and Policy Implications

When asked to rate access to current treatment services, over half of respondents (57.7%) described it as “not adequate” or “inadequate,” while only 13% considered it fully adequate. These ratings indicate substantial perceived limitations in treatment-service access among participating respondents; however, they should not be interpreted as direct evidence that clinically indicated treatment was unavailable. Perceived adequacy may reflect several dimensions of access, including availability of disease-specific expertise, geographic accessibility, rehabilitation and supportive services, administrative processes, and expectations regarding treatment options.

The most commonly reported barriers were long distances to specialized hospitals (44.1%) and lack of services at local facilities (34.7%), suggesting that geographic access and the location of specialized services were important concerns for respondents. Similar challenges have been described internationally, where the rarity of individual conditions can result in expertise being concentrated in a limited number of specialist or multidisciplinary centers [25]. Additional barriers included lack of transportation (16.9%) and inability to take time off work (16.2%), illustrating how practical and socioeconomic circumstances may interact with perceived access to care.

Disease category remained associated with perceived treatment-service adequacy in adjusted analyses accounting for current patient age, sex, and respondent type, whereas respondent type itself was not independently associated with treatment-access ratings. This suggests that some of the observed variation in treatment-access experience was not explained solely by differences in the demographic or respondent composition of the disease groups. Nevertheless, the broad disease categories include conditions with markedly different treatment options, care pathways, and need for supportive services; these differences should therefore be interpreted descriptively rather than as evidence of a uniform disease-group effect.

Respondents also expressed strong demand for home healthcare services (31.5%) and improved transition programs from pediatric to adult care (35.9%), indicating that continuity of care beyond specialist consultations was an important respondent-reported priority. Transition from pediatric to adult care is also recognized internationally as a particular challenge for people with chronic and rare conditions because continuity may be disrupted when disease-specific expertise is concentrated in pediatric services [26,27]. While 82% of patients reported regular follow-up at a health institution, 18% had no routine follow-up, the absence of routine follow-up cannot be interpreted uniformly as an unmet need, because follow-up requirements vary substantially by diagnosis, disease severity, and treatment availability.

A prominent respondent-reported priority was the establishment of specialized rare-disease centers (79.5%). This preference is consistent with international approaches that organize rare-disease care around centers of expertise and networks linking multidisciplinary specialist services [28]. Such models aim to concentrate scarce expertise while facilitating referral, consultation, and coordination across services. The present survey cannot establish whether a particular organizational model would improve outcomes in Türkiye, but the high prioritization of specialized centers indicates a strong perceived need for easier access to coordinated rare-disease expertise.

Another key finding was the mismatch between patient needs and existing social support structures. Although 50% of respondents were in contact with patient organizations, 40% were not members of any group, and some were unaware of relevant organizations altogether. Strengthening patient organizations and outreach could improve engagement, advocacy, and support with administrative processes, which respondents frequently identified as burdensome.

Over 55% reported financial difficulties, and 46.7% experienced problems obtaining or renewing disability assessment reports. Streamlining bureaucratic procedures, such as approvals for medications or disability entitlements, was supported by 74.4% of participants. Administrative processes also emerged as an important area for respondents: 74.4% identified faster completion of procedures related to access to new medications as a priority for service improvement. These findings represent respondents’ experiences and priorities and do not by themselves establish the source or extent of administrative inefficiency.

Access to medications, including orphan drugs, was another major concern: 44% reported difficulties obtaining medicines, and 67.7% identified medication access as one of the most important components of care. Medication-access responses also require cautious interpretation. The survey assessed respondents’ perceived difficulties and priorities but did not determine whether a disease-specific treatment was clinically indicated, licensed, reimbursed, or available for each individual diagnosis. Reported difficulty accessing medication should therefore be interpreted as a perceived treatment access experience rather than as direct evidence of national treatment unavailability.

Perceptions of limited medication access may also be influenced by expectations and by variation in the evidence, regulatory status, reimbursement, and commercial availability of disease-specific therapies. A requested treatment may lack sufficient evidence or may not have been submitted for regulatory approval, yet its non-reimbursement or non-availability may still be experienced and reported as an access problem [9,10]. These survey responses should therefore be interpreted as perceived access experiences rather than evaluations of the clinical appropriateness of individual treatment decisions.

Taken together, the findings on geographic access, treatment-service adequacy, transition, administrative burden, and specialized expertise are broadly consistent with challenges described in international rare-disease care [10,25,26,27,28]. At the same time, the present data are best understood as respondent-reported priorities within the Turkish healthcare context rather than as direct measures of system performance. They identify areas—particularly coordination of specialist expertise, continuity across the life course, treatment access, and administrative navigation—that warrant further evaluation using service-level and population-based data.

### 4.6. National Action Plan of Türkiye

Türkiye’s 2023–2027 Rare Diseases Health Strategy Document and Action Plan represents a significant policy step that addresses several domains that were also identified as priorities or perceived challenges by respondents in the present study [7]. The plan emphasizes earlier diagnosis, professional training, stronger referral pathways, better coordination of care, access to advanced genetic diagnostics, newborn screening expansion, collaboration with patient organizations, psychosocial support, telehealth, social services, and orphan drug access.

Several of these policy domains correspond to respondent-reported experiences in the present survey, including complex diagnostic pathways, perceived need for greater professional knowledge, psychosocial support needs, geographic barriers to specialized care, administrative difficulties, and concerns regarding access to treatment and medication. The convergence between these respondent-reported priorities and the Action Plan suggests areas in which patient and caregiver perspectives may contribute to implementation and service evaluation.

As the present study used voluntary, non-probability recruitment through patient-organization networks and a survey instrument that was not designed for repeated population-level monitoring, the findings should not be regarded as a nationally representative baseline for evaluating the Action Plan. Rather, they provide a large descriptive dataset of patient- and caregiver-reported experiences and priorities collected during the early implementation period of the national strategy. Future evaluations using reproducible sampling and standardized measures could assess changes over time in diagnostic experiences, access to specialized care, psychosocial and informational support, continuity of care, and administrative barriers.

### 4.7. Strengths and Limitations

This study has several strengths. It included a large sample of individuals and families affected by a wide range of rare diseases and captured respondents from geographically dispersed communities across Türkiye. The multidomain survey enabled diagnostic experiences, psychosocial and informational needs, functional and social consequences, healthcare access, and service priorities to be examined within the same study. The inclusion of both patient self-respondents and relatives/caregivers also provided complementary perspectives, while the respondent-type and selected multivariable sensitivity analyses allowed the potential influence of proxy reporting and case-mix differences to be explored. Nevertheless, these strengths should be considered alongside several important limitations. First, participants were recruited through rare-disease patient organizations and their communication networks using a voluntary, non-probability online approach. Although respondents were reached across Türkiye, broad geographic participation does not make the sample nationally representative. Individuals who were connected to patient organizations, had internet access, or were particularly motivated to share their experiences may have been more likely to participate, whereas individuals with limited digital access, weaker links to patient organizations, or other barriers to participation may have been underrepresented. Self-selection and online participation therefore limit generalizability of the observed proportions to the overall rare-disease population in Türkiye.

Second, rare-disease diagnoses and other clinical information were reported by respondents and were not independently verified against medical records. The anonymous survey design also precluded definitive identification of multiple questionnaires referring to the same patient. To assess this possibility, combinations of reported diagnosis, age, sex, and other patient characteristics were reviewed for potentially duplicate patient profiles, and no strongly suggestive duplicate patterns were identified. Nevertheless, duplicate participation cannot be excluded with certainty.

Third, most questionnaires were completed by relatives or caregivers rather than directly by affected individuals. Patient-level information provided by these respondents therefore represents proxy-reported information, and proxy assessments may not fully correspond to patients’ own perceptions, particularly for subjective experiences. We addressed this issue by distinguishing patient-, proxy-, respondent-, and household-level information in the manuscript and by conducting respondent-type stratified and adjusted sensitivity analyses for selected subjective outcomes. Respondent type was associated with some, but not all, outcomes, indicating that proxy perspective remains relevant to interpretation.

Fourth, several questions concerned experiences that may have occurred years before survey participation and were therefore susceptible to recall bias. This is particularly relevant to the diagnostic pathway in a sample spanning a wide age range. For this reason, the retrospectively reconstructed symptom-to-diagnosis interval is presented only as a secondary descriptive statistic and is not treated as a principal outcome. Analyses of the symptomatic diagnostic journey were instead restricted to participants diagnosed after symptom onset and focused on more directly reported indicators, including the number of physicians consulted, previous incorrect or alternative diagnoses, additional specialist opinions, and perceived possibility of an earlier diagnosis. The latter remains a subjective retrospective assessment and should not be interpreted as an objective measure of diagnostic delay.

Fifth, the survey instrument was developed specifically for this study. Although item selection was informed by previous national and international rare-disease surveys and the draft instrument was piloted with 20 participants for comprehensibility and clarity, it did not undergo formal psychometric validation. Because it was designed as a multidomain descriptive survey rather than a scale measuring a single latent construct, internal-consistency reliability was not assessed for the questionnaire as a whole. Measurement error and variation in respondents’ interpretation of subjective questions therefore remain possible. In addition, all survey items were mandatory except conditional follow up questions. Although this resulted in complete responses for applicable items, mandatory responding may have prompted some participants to select the closest available option when none fully represented their experience. In addition, several non-diagnostic items did not specify a fixed recall period, so responses may reflect experiences over different time horizons.

Sixth, individual rare diseases were grouped into broad categories to permit interpretable descriptive and exploratory comparisons. These categories inevitably contain substantial clinical heterogeneity in age of onset, disease severity, treatment availability, functional consequences, and patterns of healthcare use. The Other/unspecified category was particularly heterogeneous and relatively small; therefore, selected analyses were repeated after excluding this category as sensitivity analyses. Disease-group comparisons should nevertheless be interpreted as exploratory and not as estimates of homogeneous disease-category effects.

Seventh, the study included numerous exploratory comparisons. Formal multiplicity adjustment was not applied because these analyses were intended primarily to describe patterns and generate hypotheses rather than to test a set of prespecified confirmatory hypotheses. This increases the possibility that some statistically significant findings occurred by chance. We therefore placed greater emphasis on effect sizes, consistency of patterns, and selected adjusted analyses rather than on isolated *p* values. Sparse contingency tables were additionally evaluated for chi-square assumptions, with Monte Carlo estimates used where appropriate.

Finally, the cross-sectional design precludes causal inference. Respondent-attributed explanations for experiences or barriers cannot establish their causes; for example, employment status cannot by itself demonstrate an effect of caregiving, seeking an additional specialist opinion does not necessarily indicate dissatisfaction, and perceived difficulty accessing treatment does not establish clinical treatment unavailability. Although selected multivariable analyses accounted for age or age at diagnosis, sex, respondent type, and disease category as appropriate, residual confounding by unmeasured clinical, socioeconomic, and healthcare-related characteristics remains possible.

The survey also did not specifically assess assistive technologies. Future studies should examine their role in autonomy, communication, participation, and access to care among people living with rare diseases.

## 5. Conclusions

This study provides a broad descriptive overview of experiences and priorities reported by people living with rare diseases and their caregivers who participated in this patient-organization-recruited survey in Türkiye. Respondents described complex diagnostic journeys, including previous incorrect or alternative diagnoses, as well as psychosocial and informational needs, perceived challenges in accessing specialized care and treatment services, and effects on daily life, education, employment, finances, and social participation. Participants prioritized knowledgeable healthcare professionals, specialized centers, faster administrative processes, accurate information, medications, psychosocial support, and social services. Several of these priorities correspond to domains addressed in Türkiye’s Rare Diseases Health Strategy Document and Action Plan, suggesting areas in which patient and caregiver perspectives may inform implementation and future service evaluation. The findings may also provide a patient and caregiver informed evidence base for the development of a potential future second rare disease action plan or subsequent national policy initiatives on rare diseases in Türkiye.

The findings should not be interpreted as nationally representative estimates or as direct evidence of system-wide service deficiencies; rather, they identify respondent-reported experiences and priorities that may help guide further evaluation of coordinated, equitable, and family-centered rare-disease care. Future longitudinal and qualitative research, including repeated surveys using reproducible sampling approaches and standardized measures, could further characterize these needs and evaluate changes alongside implementation of the Action Plan.

## Figures and Tables

**Table 1:** (**A**) Respondent and patient characteristics by disease category. (**B**) Caregiver and household characteristics by disease category, %.

	Metabolic/ Storage	Neuromuscular	Neurogenetic/ Neurodegenerative	Congenital Anomaly/ Syndrome	Autoimmune/ Autoinflammatory/ Rheumatologic	Cardiovascular/ Pulmonary	Other/ Unspecified	Overall
**(A)**								
**Overall respondent type (n = 1642)**								
Self (Total)	16.5 ^a^	23.9 ^a^	18.2 ^a^	6.3 ^b^	73.4 ^c^	45.9 ^d^	60.0 ^c.d^	23.3
Caregiver (Total)	83.5 ^a^	76.1 ^a^	81.8 ^a^	93.7 ^b^	26.6 ^c^	54.1 ^d^	40.0 ^c.d^	76.7
**Sig.**	Cramer’s v/*p* value of Overall: **0.336/<0.01**; excluding other group: **0.312/<0.001**							
**Respondent type by patient’s age <18 years (n = 1091)**								
Self	1.4	1.6	0.8	0.7	0	4	0	1.4
Caregiver	98.6	98.4	99.2	99.3	100	96	100	98.6
**Sig.**	Cramer’s v/*p* value of Overall: 0.047/0.780 *; excluding other group: 0.046/0.767 *							
**Respondent type by patient’s age ≥18 years (n = 551)**								
Self	54.7 ^a^	71.2 ^b^	61.2 ^a.b^	45.0 ^a.b^	94.0 ^c^	67.3 ^a.b^	69.2 ^a.b^	66.8
Caregiver	45.3 ^a^	28.8 ^b^	38.8 ^a.b^	55.0 ^a.b^	6.0 ^c^	32.7 ^a.b^	30.8 ^a.b^	33.2
**Sig.**	Cramer’s v/*p* value of Overall: **0.244/<0.01**; excluding other group: **0.252/<0.001**							
**Patient’s age at enrollment (n = 1642)**								
<18 years	71.6 ^a^	68.0 ^a^	71.2 ^a^	87.3 ^b^	21.9 ^c^	33.8 ^c^	13.3 ^c^	66.4
≥18 years	28.4 ^a^	32.0 ^a^	28.8 ^a^	12.7 ^b^	78.1 ^c^	66.2 ^c^	86.7 ^c^	33.6
**Sig.**	Cramer’s v/*p* value of Overall: **0.339/<0.01**; excluding other group: **0.289/<0.001**							
**Patient age at diagnosis (n = 1642)**								
0–2 month	38.6 ^a^	3.1 ^b^	0.6 ^b^	20.9 ^c^	1.6 ^b^	17.6 ^c^	6.7 ^b.c^	15.8
3–11 month	14.1 ^a^	13.7 ^a^	4.1 ^b^	28.5 ^c^	6.2 ^a.b^	9.5 ^a.b^	0.0	13.4
1–4 year	30.2 ^a.b^	44.1 ^c^	45.3 ^c^	36.7 ^b.c^	6.2 ^d^	8.1 ^d^	8.9 ^a.d^	35.3
5–9 year	5.9 ^a^	17.5 ^b.c^	25.3 ^c^	8.2 ^a.b^	17.2 ^b.c^	5.4 ^a.b^	13.3 ^a.b.c^	13.3
10–17 year	5.9 ^a.b.c^	9.5 ^c.d^	13.5 ^d^	1.9 ^b^	14.1 ^a.c.d^	13.5 ^a.c.d^	22.2 ^d^	8.8
≥18 year	5.3 ^a^	12.0 ^b^	11.2 ^a.b^	3.8 ^a.b^	54.7 ^c^	45.9 ^c^	48.9 ^c^	13.3
**Sig.**	Cramer’s v/*p* value of Overall: **0.287/<0.01**; excluding other group: **0.278/<0.001**							
**Current status of patient (n = 1642)**								
Deceased	1.2	0.5	2.4	0	0	2.7	2.2	1.0
Alive	98.8	99.5	97.6	100	100	97.3	97.8	99.0
**Sig.**	Cramer’s v/*p* value of Overall: 0.080/0.103; excluding other group: 0.080/0.070							
**Sex (n = 1642)**								
Female	49.6 ^a^	22.0 ^b^	58.8 ^a^	56.3 ^a^	67.2 ^a^	63.5 ^a^	64.4 ^a^	42.1
Male	50.4 ^a^	78.0 ^b^	41.2 ^a^	43.7 ^a^	32.8 ^a^	36.5 ^a^	35.6 ^a^	57.9
**Sig.**	Cramer’s v/*p* value of Overall: **0.340/<0.01**; excluding other group: **0.337/<0.001**							
**Educational status of patient (n = 1642)**								
None	26.1 ^a^	26.7 ^a^	20.0 ^a.b^	53.8 ^c^	6.2 ^b^	12.2 ^a.b^	2.2 ^b^	26.3
Primary or less	42.9 ^a.b^	45.7 ^a.b^	51.8 ^b^	32.9 ^a.c^	21.9 ^c^	28.4 ^a.c^	15.6 ^c^	41.7
High school	18.4 ^a.b^	14.7 ^a.b^	17.6 ^a.b^	8.2 ^b^	23.4 ^a^	20.3 ^a.b^	17.8 ^a.b^	16.1
University	12.7 ^a^	12.9 ^a^	10.6 ^a^	5.1 ^a^	48.4 ^b^	39.2 ^b^	64.4 ^b^	15.8
**Sig.**	Cramer’s v/*p* value of Overall: **0.233/<0.01**; excluding other group: **0.198/<0.001**							
**(B)**								
**Educational status of caregiver (n = 1642)**								
No caregiver	15.1 ^a^	10.9 ^a.b^	7.6 ^a.b^	5.7 ^b^	54.7 ^c^	37.8 ^c^	44.4 ^c^	15.2
Primary or less	31.4 ^a.b.c.d^	37.8 ^c.d^	37.1 ^b.d^	11.4 ^e^	12.5 ^e^	16.2 ^a.e^	22.2 ^a.b.c.d.e^	30.9
High school	24.1 ^a.b^	25.6 ^b^	25.3 ^b^	29.7 ^b^	14.1 ^a.b^	21.6 ^a.b^	4.4 ^a^	24.3
University or more	29.4 ^a^	25.7 ^a^	30.0 ^a^	53.2 ^b^	18.8 ^a^	24.3 ^a^	28.9 ^a.b^	29.7
**Sig.**	Cramer’s v/*p* value of Overall: **0.218/<0.01**; excluding other group: **0.207/<0.001**							
**Working status of patient (n = 1642)**								
Not of working age	44.7 ^a^	46.5 ^a^	39.4 ^a.b^	67.1 ^c^	10.9 ^d^	24.3 ^b.d^	6.7 ^d^	43.7
Unable to work	11.6 ^a^	22.6 ^b.c^	28.2 ^c^	4.4 ^a^	7.8 ^a.b^	10.8 ^a.b.c^	15.6 ^a.b.c^	16.9
Able to work but unemployed	3.9 ^a^	3.6 ^a^	5.9 ^a^	1.9 ^a^	7.8 ^a^	5.4 ^a^	8.9 ^a^	4.1
Housewife	2.9 ^a^	2.5 ^a^	1.8 ^a^	3.2 ^a.b^	14.1 ^b^	6.8 ^a.b^	0.0	3.2
Student	26.7 ^a^	16.5 ^b^	15.9 ^a.b^	18.4 ^a.b^	15.6 ^a.b^	20.3 ^a.b^	20.0 ^a.b^	19.9
Public sector employee	4.1 ^a^	5.5 ^a.b^	6.5 ^a.b^	1.9 ^a^	14.1 ^b.c^	9.5 ^a.b.c^	22.2 ^c^	5.8
Private sector employee	5.1 ^a^	0.9 ^b^	0.6 ^a.b^	2.5 ^a.b^	15.6 ^c^	5.4 ^a.c^	17.8 ^c^	3.5
Retired	1.0 ^a^	1.9 ^a.b^	1.8 ^a.b^	0.6 ^a^	14.1 ^c^	17.6 ^c^	8.9 ^b.c^	2.9
**Sig.**	Cramer’s v/*p* value of Overall: 0.196/<0.001 *; excluding other group: 0.199/<0.001 *							
**Working status of caregiver (n = 1642)**								
No caregiver	23.3 ^a^	18.3 ^a^	13.5 ^a^	19.0 ^a^	67.2 ^b^	43.2 ^b^	51.1 ^b^	23.3
Unable to work	5.1 ^a^	4.4 ^a^	2.4 ^a^	2.5 ^a^	3.1 ^a^	0.0	0.0	3.8
Able to work but unemployed	4.1 ^a^	3.4 ^a^	5.3 ^a^	1.9 ^a^	0.0	0.0	2.2 ^a^	3.4
Housewife	52.1 ^a^	53.2 ^a^	59.4 ^a^	43.0 ^a.b^	10.9 ^c^	25.7 ^b.c^	22.2 ^b.c^	48.8
Student	0.2 ^a^	0.9 ^a^	0.0	1.3 ^a^	0.0	1.4 ^a^	2.2 ^a^	0.7
Public sector employee	6.7 ^a^	6.1 ^a^	8.2 ^a.b^	17.1 ^b^	1.6 ^a^	12.2 ^a.b^	2.2 ^a.b^	7.6
Private sector employee	4.7 ^a^	6.4 ^a^	7.1 ^a^	7.0 ^a^	12.5 ^a^	8.1 ^a^	4.4 ^a^	6.3
Retired	2.9 ^a^	5.1 ^a.b^	1.8 ^a^	5.7 ^a.b^	4.7 ^a.b^	9.5 ^a.b^	13.3 ^b^	4.6
Not working to care for child	0.8 ^a^	2.2 ^a^	2.4 ^a^	2.5 ^a^	0.0	0.0	2.2 ^a^	1.6
**Sig.**	Cramer’s v/*p* value of Overall: 0.150/<0.001 *; excluding other group: 0.154/<0.001 *							
**Total monthly household income (n = 1642)**								
<17.000	30.4 ^a^	30.9 ^a^	27.1 ^a^	12.0 ^b^	17.2 ^a.b^	24.3 ^a.b^	20.0 ^a.b^	27.4
17.000–35.000	38.0 ^a^	43.5 ^a^	42.9 ^a^	40.5 ^a^	28.1 ^a^	29.7 ^a^	33.3 ^a^	40.0
35.000–50.000	19.2 ^a.b^	15.3 ^b^	18.8 ^a.b^	21.5 ^a.b^	29.7 ^a.b^	33.8 ^a^	22.2 ^a.b^	19.0
>50.000	12.4 ^a.b^	10.3 ^b^	11.2 ^a.b^	25.9 ^c^	25.0 ^a.c^	12.2 ^a.b.c^	24.4 ^a.b.c^	13.6
**Sig.**	Cramer’s v/*p* value of Overall: **0.128/<0.01**; excluding other group: **0.126/<0.001**							

Bold *p* values denote statistical significance. Superscript values in the same row that do not share a letter differ at *p* < 0.05. Values are column percentages; denominators for each disease category are pro-vided in the column headings. *p* values are based on Pearson’s chi-square tests unless otherwise specified. Cramér’s V is reported as the effect size for categorical comparisons. Monte Carlo estimates were used when expected-cell assumptions for the chi-square test were not satisfied (shown with *). Each last row of question groups presents both overall analysis and a sensitivity analysis after exclusion of the Other/unspecified disease category.

**Table 2:** Family history, information sources, follow-up, and support by disease category, %.

	Metabolic/ Storage	Neuromuscular	Neurogenetic/ Neurodegenerative	Congenital Anomaly/ Syndrome	Autoimmune/ Autoinflammatory/ Rheumatologic	Cardiovascular/ Pulmonary	Other/ Unspecified	Overall	Cramer’s V, *p* Value	Cramer’s V, *p* Value * Excluding Others Group
**Presence of same rare disease in family (n = 1642)**										
Single case	75.7 ^a^	72.1 ^a.b^	64.7 ^a.b^	96.2 ^c^	56.2 ^b^	82.4 ^a^	75.6 ^a.b^	74.7	**0.198**, **<0.01**	**0.200**, **<0.001**
2 or more case	24.3 ^a^	27.9 ^a.b^	35.3 ^a.b^	3.8 ^c^	43.8 ^b^	17.6 ^a^	24.4 ^a.b^	25.3		
**Awareness of the disease before diagnosis (n = 1642)**										
No, I heard it when diagnosed	86.7 ^a.b^	84.1 ^b^	91.8 ^a.b^	94.3 ^a^	82.8 ^a.b^	91.9 ^a.b^	91.1 ^a.b^	87.1	**0.108**, **0.004**	**0.107**, **0.003**
Yes, I had heard of it	13.3 ^a.b^	15.9 ^b^	8.2 ^a.b^	5.7 ^a^	17.2 ^a.b^	8.1 ^a.b^	8.9 ^a.b^	12.9		
**Sources utilized to obtain information about the disease (n = 1642)**										
None	1.8	1.4	2.4	3.2	3.1	1.4	0.0	1.8	0.049, 0.670 *	0.044, 0.694 *
Internet—Social Media	78.8	82.2	84.1	81.6	85.9	83.8	73.3	81.3	0.062, 0.380	0.053, 0.480
Health professionals	55.3 ^a^	47.3 ^a.b^	40.6 ^b^	49.4 ^a.b^	57.8 ^a.b.c^	75.7 ^c^	62.2 ^a.b.c^	51.3	**0.148**, **<0.01**	**0.146**, **<0.001**
Patient organizations	50.8 ^a.b^	65.8 ^c^	32.4 ^d^	35.4 ^d^	48.4 ^a.b.c.d^	66.2 ^b.c^	35.6 ^a.d^	53.5	**0.251**, **<0.01**	**0.247**, **<0.001**
Individuals with same disease	63.5 ^a^	62.1 ^a^	67.6 ^a^	73.4 ^a^	37.5 ^b^	62.2 ^a.b^	57.8 ^a.b^	63.1	**0.130**, **<0.01**	**0.130**, **<0.001**
**Presence of routine follow-ups in a health institution (n = 1642)**										
No	10.8 ^a^	24.6 ^b^	25.9 ^b^	11.4 ^a^	9.4 ^a.b^	4.1 ^a^	20.0 ^a.b^	17.7	**0.194**, **<0.01**	**0.197**, **<0.001**
Yes	89.2 ^a^	75.4 ^b^	74.1 ^b^	88.6 ^a^	90.6 ^a.b^	95.9 ^a^	80.0 ^a.b^	82.3		
**Perceived need for emotional, psychosocial, or psychiatric support at or after the time of diagnosis (n = 1642)**										
No	25.9 ^a.b.c^	27.5 ^c^	24.7 ^a.b.c^	14.6 ^b^	37.5 ^a.c^	24.3 ^a.b.c^	28.9 ^a.b.c^	25.8	**0.100**, **0.012**	**0.101**, **0.006**
Yes	74.1 ^a.b.c^	72.5 ^c^	75.3 ^a.b.c^	85.4 ^b^	62.5 ^a.c^	75.7 ^a.b.c^	71.1 ^a.b.c^	74.2		
**Previous receipt of psychosocial support from health professionals (n = 1642)**										
No	68.4 ^a^	69.7 ^a^	59.4 ^a.b^	51.3 ^b^	53.1 ^a.b^	66.2 ^a.b^	62.2 ^a.b^	65.5	**0.131**, **<0.01**	**0.133**, **<0.001**
Yes	31.6 ^a^	30.3 ^a^	40.6 ^a.b^	48.7 ^b^	46.9 ^a.b^	33.8 ^a.b^	37.8 ^a.b^	34.5		
**Membership in a patient association or non-governmental organization related to the disease (n = 1642)**										
No membership	41.0 ^a^	35.9 ^a^	47.6 ^a^	44.9 ^a^	50.0 ^a^	40.5 ^a^	44.4 ^a^	40.5	**0.194**, **<0.01**	**0.182**, **<0.001**
Yes, I have membership/contact	51.0 ^a.b.c^	60.1 ^c^	32.9 ^d^	39.2 ^a.b.d^	28.1 ^d^	58.1 ^b.c^	28.9 ^a.d^	50.4		
Can’t find/don’t know	8.0 ^a.b.c^	4.1 ^c^	19.4 ^d^	15.8 ^b.d^	21.9 ^d^	1.4 ^a.c^	26.7 ^d^	9.1		

Bold *p* values denote statistical significance. Superscript values in the same row that do not share a letter differ at *p* < 0.05. Values are column percentages; denominators for each disease category are pro-vided in the column headings. *p* values are based on Pearson’s chi-square tests unless otherwise specified. Cramér’s V is reported as the effect size for categorical comparisons. Monte Carlo estimates were used when expected-cell assumptions for the chi-square test were not satisfied (shown with *). Each last row of question groups presents both overall analysis and a sensitivity analysis after exclusion of the Other/unspecified disease category. For multiple-response items, participants could select more than one option; therefore, percentages may exceed 100%.

**Table 3:** (**A**) Disease-related functional difficulties by disease category. (**B**) Consequences experienced following diagnosis by disease category. (**C**) Ongoing disease-related challenges by disease category, %.

	Metabolic/ Storage	Neuromuscular	Neurogenetic/ Neurodegenerative	Congenital Anomaly/ Syndrome	Autoimmune/ Autoinflammatory/ Rheumatologic	Cardiovascular/ Pulmonary	Other/ Unspecified	Overall	Cramer’s V, *p* Value	Cramer’s V, *p* Value * Excluding Others Group
**(A)**										
**Major difficulties experienced due to the disease**										
None	9.6 ^a^	8.7 ^a^	2.9 ^a^	3.8 ^a^	12.5 ^a^	10.8 ^a^	11.1 ^a^	8.2	**0.094**, **0.024**	**0.094**, **0.015**
Difficulties in performing daily activities	38.4 ^a^	53.5 ^b.c^	65.3 ^c^	46.2 ^a.b^	54.7 ^a.b.c^	56.8 ^a.b.c^	53.3 ^a.b.c^	49.7	**0.172**, **<0.001**	**0.174**, **<0.001**
Challenges in self-care abilities	32.0 ^a^	59.6 ^b^	73.5 ^c^	54.4 ^b.d^	29.7 ^a^	37.8 ^a.d^	26.7 ^a^	49.3	**0.302**, **<0.001**	**0.297**, **<0.001**
Limitations due to loss of mobility or sensory functions	28.2 ^a.b^	43.4 ^c^	65.9 ^d^	29.1 ^a.b^	28.1 ^a.b.c^	14.9 ^b^	40.0 ^a.c^	37.8	**0.258**, **<0.001**	**0.261**, **<0.001**
Difficulties in academic or occupational functioning	53.7 ^a.b^	47.1 ^b.c^	62.4 ^a^	51.3 ^a.b.c^	57.8 ^a.b.c^	33.8 ^c^	48.9 ^a.b.c^	50.9	**0.121**, **0.001**	**0.123**, **<0.001**
Challenges in learning and comprehension	26.5 ^a.b^	17.6 ^c^	38.8 ^b^	57.6 ^d^	12.5 ^a.c^	10.8 ^a.c^	24.4 ^a.b.c^	26.0	**0.287**, **<0.001**	**0.290**, **<0.001**
Limitations in social life and relationships	51.2 ^a^	51.3 ^a^	67.6 ^b^	66.5 ^b^	51.6 ^a.b^	51.4 ^a.b^	53.3 ^a.b^	54.5	**0.126**, **<0.001**	**0.128**, **<0.001**
Difficulties in communication with others	16.9 ^a^	16.2 ^a^	38.2 ^b^	32.3 ^b^	9.4 ^a^	10.8 ^a^	24.4 ^a.b^	20.0	**0.203**, **<0.001**	**0.206**, **<0.001**
Challenges in behavioral regulation	14.1 ^a^	13.9 ^a^	26.5 ^b.c^	34.8 ^c^	9.4 ^a.b^	9.5 ^a.b^	24.4 ^a.b.c^	17.2	**0.192**, **<0.001**	**0.193**, **<0.001**
**(B)**										
**Challenges experienced following the diagnosis (n = 1642)**										
None	19.4 ^a^	19.7 ^a^	11.2 ^a^	18.4 ^a^	17.2 ^a^	14.9 ^a^	13.3 ^a^	18.1	0.071, 0.213	0.069, 0.180
Resignation from employment due to the condition	9.8 ^a^	12.5 ^a.b^	10.0 ^a.b^	19.6 ^b^	6.2 ^a.b^	13.5 ^a.b^	22.2 ^a.b^	12.2	**0.105**, **0.006**	**0.094**, **0.016**
Reduced employment opportunities	16.7 ^a^	17.6 ^a.b^	18.8 ^a.b^	18.4 ^a.b^	32.8 ^b^	10.8 ^a^	26.7 ^a.b^	18.1	**0.096**, **0.020**	**0.090**, **0.025**
Relocation required due to hospital access or related reasons	14.3 ^a^	17.3 ^a^	14.7 ^a^	14.6 ^a^	14.1 ^a^	18.9 ^a^	13.3 ^a^	15.7	0.044, 0.785	0.043, 0.703
Deterioration in financial situation	44.7 ^a^	36.5 ^a^	35.9 ^a^	38.6 ^a^	25.0 ^a^	28.4 ^a^	22.2 ^a^	37.9	**0.117**, **0.001**	**0.105**, **0.003**
Restricted social life	63.3 ^a.b^	61.2 ^b^	74.7 ^a^	67.7 ^a.b^	67.2 ^a.b^	71.6 ^a.b^	71.1 ^a.b^	64.8	**0.094**, **0.024**	**0.093**, **0.017**
Weakened contact with relatives/friends due to the disease	47.6 ^a^	53.2 ^a.b^	64.7 ^b^	53.2 ^a.b^	35.9 ^a^	58.1 ^a.b^	46.7 ^a.b^	52.1	**0.120**, **0.001**	**0.120**, **<0.001**
**(C)**										
**Main challenges experienced in relation to the disease**										
No disease-related difficulties were experienced	0.4 ^a^	1.9 ^a^	0	1.3 ^a^	1.6 ^a^	1.4 ^a^	2.2 ^a^	1.2	0.069, 0.228 *	0.069, 0.167 *
Difficulties during the diagnostic process	48.0 ^a^	58.3 ^b^	62.4 ^b^	58.9 ^a.b^	73.4 ^b^	63.5 ^a.b^	48.9 ^a.b^	56.3	**0.131**, **<0.001**	**0.130**, **<0.001**
Frequent requirement for medical testing	49.0 ^a^	33.1 ^b^	37.6 ^a.b^	39.9 ^a.b^	54.7 ^a^	50.0 ^a.b^	33.3 ^a.b^	40.6	**0.154**, **<0.001**	**0.154**, **<0.001**
Challenges in accessing healthcare facilities	44.3 ^a^	43.5 ^a^	35.9 ^a^	31.6 ^a^	35.9 ^a^	37.8 ^a^	35.6 ^a^	41.0	**0.089**, **0.044**	**0.088**, **0.030**
Frequent need for hospitalization	21.4 ^a^	10.0 ^b^	18.8 ^a^	28.5 ^a^	20.3 ^a.b^	25.7 ^a^	17.8 ^a.b^	17.4	**0.170**, **<0.001**	**0.173**, **<0.001**
Limited access to specialist physicians	36.1 ^a^	43.1 ^a^	42.9 ^a^	41.8 ^a^	50.0 ^a^	36.5 ^a^	35.6 ^a^	40.6	0.076, 0.148	0.075, 0.109
Problems in accessing medications	46.9 ^a^	50.2 ^a^	40.6 ^a^	20.9 ^b^	34.4 ^a.b^	44.6 ^a^	37.8 ^a.b^	44.2	**0.174**, **<0.001**	**0.175**, **<0.001**
Difficulties in receiving home healthcare services	11.0 ^a^	19.5 ^b^	17.6 ^a.b^	8.2 ^a^	7.8 ^a.b^	12.2 ^a.b^	11.1 ^a.b^	14.7	**0.128**, **<0.001**	**0.128**, **<0.001**
Special nutritional requirements	48.4 ^a^	24.2 ^b^	22.9 ^b^	35.4 ^a.b^	26.6 ^b^	20.3 ^b^	15.6 ^b^	32.0	**0.243**, **<0.001**	**0.238**, **<0.001**
Challenges in communicating with patient organizations	11.4 ^a^	14.0 ^a^	11.8 ^a^	17.1 ^a^	4.7 ^a^	5.4 ^a^	13.3 ^a^	12.5	0.085, 0.062	**0.087**, **0.035**
Feelings of uncertainty or confusion regarding what to do	60.4 ^a^	62.2 ^a^	70.0 ^a.b^	80.4 ^b^	51.6 ^a^	56.8 ^a^	57.8 ^a^	63.5	**0.137**, **<0.001**	**0.138**, **<0.001**
Difficulties in educational or occupational life	33.9 ^a^	38.4 ^a.b^	48.8 ^b^	41.8 ^a.b^	51.6 ^a.b^	40.5 ^a.b^	57.8 ^b^	39.6	**0.120**, **0.001**	**0.104**, **0.004**
Financial difficulties	61.0 ^a^	54.9 ^a.b^	50.6 ^a.b^	58.2 ^a.b^	37.5 ^b^	52.7 ^a.b^	37.8 ^a.b^	55.4	**0.117**, **0.001**	**0.103**, **0.005**
Limited access to medical equipment	14.3 ^a^	40.1 ^b^	25.9 ^c.d.e^	16.5 ^a.e^	6.2 ^a^	40.5 ^b.d^	13.3 ^a.c.e^	26.6	**0.283**, **<0.001**	**0.281**, **<0.001**
Problems in communication with government institutions	29.6 ^a^	37.3 ^a^	32.4 ^a^	37.3 ^a^	23.4 ^a^	32.4 ^a^	31.1 ^a^	33.6	0.084, 0.070	**0.085**, **0.042**
Difficulties in obtaining or renewing disability report	42.7 ^a.b.c.d^	50.7 ^c.d.e^	52.9 ^b.d.e^	58.9 ^e^	18.8 ^f^	29.7 ^a.f^	35.6 ^a.b.c.d.e.f^	46.7	**0.175**, **<0.001**	**0.173**, **<0.001**

Bold *p* values denote statistical significance. Superscript values in the same row that do not share a letter differ at *p* < 0.05. Values are column percentages; denominators for each disease category are provided in the column headings. *p* values are based on Pearson’s chi-square tests unless otherwise specified. Cramér’s V is reported as the effect size for categorical comparisons. Monte Carlo estimates were used when expected-cell assumptions for the chi-square test were not satisfied (shown with *). The final column presents a sensitivity analysis after exclusion of the Other/unspecified disease category. For multiple-response items, participants could select more than one option; therefore, percentages may exceed 100%.

**Table 4:** (**A**) Diagnostic timing in the full sample. (**B**) Symptomatic diagnostic journey among participants diagnosed after symptom onset. (**C**) Perceived reasons for delayed diagnosis, %.

	Metabolic/ Storage	Neuromuscular	Neurogenetic/ Neurodegenerative	Congenital Anomaly/ Syndrome	Autoimmune/ Autoinflammatory/ Rheumatologic	Cardiovascular/ Pulmonary	Other/ Unspecified	Overall	Cramer’s V, *p* Value	Cramer’s V, *p* Value * Excluding Others Group
**(A)**										
**Diagnostic timing (n = 1642)**										
Prenatal diagnosis	0.0 ^a^	0.0 ^a^	0.0 ^a^	6.3 ^b^	0.0 ^a.b^	0.0 ^a.b^	0.0 ^a.b^	0.6	**0.278**, <0.001 *	**0.276**, <0.001 *
Diagnosis through newborn screening at birth	26.5 ^a^	1.6 ^b^	0.0 ^b^	0.0 ^b^	0.0 ^b^	13.5 ^a.c^	0.0 ^b.c^	9.1		
Diagnosis before symptom onset	0.6 ^a^	3.6 ^b^	0.6 ^a.b^	1.9 ^a.b^	0.0 ^a.b^	0.0 ^a.b^	2.2 ^a.b^	1.9		
Diagnosis after symptom onset	65.7 ^a^	94.9 ^b.c^	98.8 ^b^	75.3 ^a.d^	98.4 ^b.c^	86.5 ^c.d^	97.8 ^b.c^	84.5		
Diagnosis by clinical examination at birth	7.1 ^a^	0.0 ^b^	0.6 ^b.c^	16.5 ^d^	1.6 ^a.c^	0.0 ^a.b.c^	0.0 ^a.b.c.d^	3.8		
**(B)**										
**Number of physicians consulted between symptom onset and definitive diagnosis (n = 1388)**										
One physician	13.0 ^a^	9.7 ^a^	7.7 ^a^	9.2 ^a^	1.6 ^a^	7.8 ^a^	4.5 ^a^	9.6	**0.131**, **<0.001**	**0.126**, **<0.001**
2–3 physicians	37.6 ^a^	51.8 ^b^	39.3 ^a.b^	40.3 ^a.b^	20.6 ^a^	43.8 ^a.b^	34.1 ^a.b^	43.7		
4–10 physicians	33.9 ^a.b^	28.1 ^a^	36.9 ^a.b^	31.9 ^a.b^	33.3 ^a.b^	34.4 ^a.b^	52.3 ^b^	32.1		
11–20 physicians	9.3 ^a^	6.6 ^a^	9.5 ^a^	7.6 ^a^	25.4 ^b^	9.4 ^a.b^	6.8 ^a.b^	8.6		
>20 physicians	6.2 ^a.b^	3.8 ^a^	6.5 ^a.b.c^	10.9 ^b.c^	19.0 ^c^	4.7 ^a.b.c^	2.3 ^a.b.c^	6.0		
**History of receiving an incorrect or alternative diagnosis before confirmation (n = 1388)**										
No	67.4 ^a.b^	72.7 ^b^	58.9 ^a^	70.6 ^a.b^	22.2 ^c^	51.6 ^a^	47.7 ^a.c^	65.6	**0.244**, **<0.001**	**0.240**, **<0.001**
Yes	32.6 ^a.b^	27.3 ^b^	41.1 ^a^	29.4 ^a.b^	77.8 ^c^	48.4 ^a^	52.3 ^a.c^	34.4		
**Seeking a second opinion from another specialist following the initial diagnosis (n = 1388)**										
None	44.1 ^a^	30.4 ^b^	34.5 ^a.b^	52.1 ^a^	34.9 ^a.b^	35.9 ^a.b^	18.2 ^b^	36.0	**0.118**, **<0.001**	**0.104**, **<0.001**
One additional specialist	26.4 ^a^	26.3 ^a^	25.0 ^a^	19.3 ^a^	20.6 ^a^	23.4 ^a^	22.7 ^a^	25.1		
Two additional specialists	11.8 ^a^	22.5 ^b^	23.2 ^b^	10.9 ^a.b^	22.2 ^a.b^	17.2 ^a.b^	18.2 ^a.b^	18.7		
Three or more additional specialists	17.7 ^a^	20.7 ^a^	17.3 ^a^	17.6 ^a^	22.2 ^a.b^	23.4 ^a.b^	40.9 ^b^	20.2		
**Perception that the diagnosis could have been made earlier (n = 1388)**										
No	20.5 ^a^	26.5 ^a^	19.6 ^a^	22.7 ^a^	14.3 ^a^	18.8 ^a^	20.5 ^a^	22.8	0.085, 0.128	0.085, 0.083
Yes	79.5 ^a^	73.5 ^a^	80.4 ^a^	77.3 ^a^	85.7 ^a^	81.2 ^a^	79.5 ^a^	77.2		
**(C)**										
**Perceived reasons for delayed diagnosis (n = 1642)**										
Difficulty accessing healthcare facilities	9.6 ^a^	10.0 ^a^	8.2 ^a^	4.4 ^a^	6.2 ^a^	6.8 ^a^	6.7 ^a^	8.8	0.063, 0.369	0.062, 0.286
Difficulty obtaining referral to the appropriate specialty	21.8 ^a^	18.9 ^a^	18.2 ^a^	20.3 ^a^	26.6 ^a^	25.7 ^a^	22.2 ^a^	20.5	0.054, 0.563	0.055, 0.444
Healthcare professionals’ lack of disease knowledge	42.4 ^a^	46.6 ^a.b^	63.5 ^c^	57.0 ^b.c^	71.9 ^c^	55.4 ^a.b.c^	62.2 ^a.b.c^	49.9	**0.166**, **<0.001**	**0.163**, **<0.001**
Diagnostic tests unavailable	33.9 ^a^	44.8 ^b^	45.9 ^a.b^	38.0 ^a.b^	50.0 ^a.b^	40.5 ^a.b^	46.7 ^a.b^	41.0	**0.108**, **0.004**	**0.108**, **0.002**
Diagnostic testing conducted too late	25.5 ^a^	31.0 ^a.b^	40.0 ^b^	23.4 ^a^	39.1 ^a.b^	25.7 ^a.b^	17.8 ^a.b^	29.3	**0.118**, **<0.001**	**0.111**, **<0.001**

Bold *p* values denote statistical significance. Superscript values in the same row that do not share a letter differ at *p* < 0.05. Values are column percentages; denominators for each disease category are provided in the column headings. *p* values are based on Pearson’s chi-square tests unless otherwise specified. Cramér’s V is reported as the effect size for categorical comparisons. Monte Carlo estimates were used when expected-cell assumptions for the chi-square test were not satisfied (shown with *). The final column presents a sensitivity analysis after exclusion of the Other/unspecified disease category. For multiple-response items, participants could select more than one option; therefore, percentages may exceed 100%.

**Table 5:** (**A**) Perceived information and communication gaps by disease category. (**B**) Perceived adequacy of and barriers to treatment-service access by disease category. (**C**) Important components of diagnosis and treatment by disease category. (**D**) Priorities for improving diagnostic and treatment services by disease category, %.

	Metabolic/ Storage	Neuromuscular	Neurogenetic/ Neurodegenerative	Congenital Anomaly/ Syndrome	Autoimmune/ Autoinflammatory/ Rheumatologic	Cardiovascular/ Pulmonary	Other/ Unspecified	Overall	Cramer’s V, *p* Value	Cramer’s V, *p* Value Excluding Others Group
**(A)**										
**Perceived gaps in disease-related information and communication (n = 1642)**										
No deficiencies were perceived	21.6 ^a^	14.4 ^b^	17.1 ^a.b^	9.5 ^b^	21.9 ^a.b^	29.7 ^a^	20.0 ^a.b^	17.5	**0.126**, **<0.001**	**0.127**, **<0.001**
Manner of communication	25.1 ^a^	33.1 ^a^	30.6 ^a^	25.3 ^a^	20.3 ^a^	27.0 ^a^	26.7 ^a^	28.7	**0.088**, **0.049**	**0.089**, **0.028**
Lack of support or counseling services	44.5 ^a^	49.5 ^a^	46.5 ^a^	51.9 ^a^	42.2 ^a^	39.2 ^a^	53.3 ^a^	47.3	0.067, 0.284	0.065, 0.241
Insufficient information provided about the disease	47.1 ^a^	59.6 ^b^	61.8 ^b^	67.7 ^b^	51.6 ^a.b^	36.5 ^a^	60.0 ^a.b^	55.5	**0.160**, **<0.001**	**0.162**, **<0.001**
Limited time spent with the healthcare professional	45.1 ^a.b^	52.6 ^b^	36.5 ^a^	44.3 ^a.b^	53.1 ^a.b^	51.4 ^a.b^	40.0 ^a.b^	47.5	**0.107**, **0.004**	**0.106**, **0.003**
**(B)**										
**Perceived adequacy of access to current treatment services for the disease (n = 1642)**										
Not adequate	22.2 ^a^	39.6 ^b^	31.2 ^a.b^	27.8 ^a.b^	23.4 ^a.b^	23.0 ^a.b^	26.7 ^a.b^	30.7	**0.138**, **<0.001**	**0.140**, **<0.001**
Inadequate	25.1 ^a^	29.5 ^a^	28.8 ^a^	24.1 ^a^	23.4 ^a^	23.0 ^a^	26.7 ^a^	27.0		
Moderately adequate	32.7 ^a^	24.2 ^b^	31.8 ^a.b^	32.9 ^a.b^	35.9 ^a.b^	29.7 ^a.b^	33.3 ^a.b^	29.3		
Adequate	20.0 ^a^	6.7 ^b^	8.2 ^b.c^	15.2 ^a.c^	17.2 ^a.b.c^	24.3 ^a^	13.3 ^a.b.c^	13.0		
**Perceived barriers to access treatment services (n = 1642)**										
Long distance to the hospital	46.3 ^a^	47.6 ^a^	34.7 ^a^	34.8 ^a^	43.8 ^a^	48.6 ^a^	31.1 ^a^	44.1	**0.109**, **0.003**	**0.101**, **0.006**
Presence of another individual at home requiring care	6.7 ^a^	10.9 ^a.b^	16.5 ^b^	7.0 ^a.b^	6.2 ^a.b^	4.1 ^a.b^	8.9 ^a.b^	9.3	**0.111**, **0.002**	**0.113**, **0.001**
Lack of transportation to reach the hospital	11.4 ^a^	21.4 ^b^	25.3 ^b^	10.1 ^a^	9.4 ^a.b^	18.9 ^a.b^	11.1 ^a.b^	16.9	**0.150**, **<0.001**	**0.150**, **<0.001**
Inability to get leave from work	19.4 ^a^	13.9 ^a^	13.5 ^a^	19.0 ^a^	20.3 ^a^	12.2 ^a^	15.6 ^a^	16.2	0.077, 0.138	0.078, 0.085
Uncertainty about where or which department to apply	7.6 ^a^	11.7 ^a.b^	11.8 ^a.b^	15.8 ^b^	14.1 ^a.b^	4.1 ^a.b^	11.1 ^a.b^	10.6	**0.094**, **0.024**	**0.096**, **0.012**
Required healthcare services not being provided	24.5 ^a^	46.5 ^b^	32.4 ^a^	30.4 ^a^	26.6 ^a^	21.6 ^a^	35.6 ^a.b^	34.7	**0.208**, **<0.001**	**0.211**, **<0.001**
**(C)**										
**Most important components during the diagnosis and treatment of the disease (n = 1642)**										
Access to accurate information about the disease	72.4 ^a^	79.1 ^a.b^	74.7 ^a^	88.0 ^b^	79.7 ^a.b^	74.3 ^a.b^	71.1 ^a.b^	77.1	**0.111**, **0.003**	**0.110**, **0.002**
Access to appropriate medical specialists or healthcare professionals	71.0 ^a^	75.4 ^a^	70.0 ^a^	86.7 ^b^	85.9 ^a.b^	86.5 ^a.b^	77.8 ^a.b^	75.6	**0.130**, **<0.001**	**0.131**, **<0.001**
Access to medications	66.9 ^a.b.c^	77.8 ^d^	60.0 ^c^	38.0 ^e^	64.1 ^a.b.c.d^	82.4 ^b.d^	46.7 ^a.c.e^	67.7	**0.265**, **<0.001**	**0.259**, **<0.001**
Access to medical devices	25.3 ^a^	58.8 ^b^	34.7 ^a^	26.6 ^a^	20.3 ^a^	66.2 ^b^	24.4 ^a^	41.1	**0.337**, **<0.001**	**0.336**, **<0.001**
Communication with other patients or patient groups	46.9 ^a^	49.3 ^a^	50.0 ^a^	70.3 ^b^	42.2 ^a^	45.9 ^a^	55.6 ^a.b^	50.4	**0.136**, **<0.001**	**0.137**, **<0.001**
Awareness of new treatment trials related to the disease	69.2 ^a^	76.8 ^a^	78.2 ^a^	71.5 ^a^	62.5 ^a^	71.6 ^a^	68.9 ^a^	73.1	**0.095**, **0.022**	**0.095**, **0.013**
Utilization of home healthcare services	20.4 ^a^	41.8 ^b^	44.1 ^b^	19.0 ^a^	18.8 ^a^	35.1 ^a.b^	13.3 ^a^	31.5	**0.241**, **<0.001**	**0.235**, **<0.001**
**(D)**										
**Key elements to improve quality of diagnostic and treatment services for disease**										
Establishment/Presence of specialized centers for rare diseases	74.1 ^a.b^	84.9 ^c^	77.6 ^a.b.c^	81.6 ^a.b.c^	70.3 ^a.b.c^	86.5 ^b.c^	62.2 ^a^	79.5	**0.145**, **<0.001**	**0.129**, **<0.001**
Increasing healthcare professionals’ knowledge about the disease	74.9 ^a^	86.3 ^b^	81.2 ^a.b^	95.6 ^c^	82.8 ^a.b^	86.5 ^a.b.c^	77.8 ^a.b^	82.9	**0.169**, **<0.001**	**0.171**, **<0.001**
Improving communication between patients and healthcare providers	63.3 ^a.b^	68.2 ^a.b^	50.0 ^c^	73.4 ^b^	51.6 ^a.c^	68.9 ^a.b.c^	73.3 ^a.b.c^	64.9	**0.140**, **<0.001**	**0.138**, **<0.001**
Facilitation of the transition from pediatric to adult care	33.9 ^a^	41.0 ^a^	32.4 ^a^	31.0 ^a^	29.7 ^a^	37.8 ^a^	20.0 ^a^	35.9	**0.101**, **0.010**	**0.086**, **0.038**
Being informed about and accessing available social services	58.4 ^a^	61.3 ^a.b^	60.6 ^a.b^	73.4 ^b^	62.5 ^a.b^	56.8 ^a.b^	55.6 ^a.b^	61.2	**0.089**, **0.045**	**0.088**, **0.031**
Acceleration of bureaucratic processes to access new medications	66.3 ^a^	88.8 ^b^	72.9 ^a^	42.4 ^c^	70.3 ^a.d^	90.5 ^b.d^	55.6 ^a.c^	74.4	**0.341**, **<0.001**	**0.340**, **<0.001**
More effective use of family physician services	35.1 ^a.b^	36.8 ^a.b^	30.0 ^b^	34.2 ^a.b^	53.1 ^a^	43.2 ^a.b^	35.6 ^a.b^	36.2	**0.089**, **0.043**	**0.090**, **0.023**

Bold *p* values denote statistical significance. Superscript values in the same row that do not share a letter differ at *p* < 0.05. Values are column percentages; denominators for each disease category are provided in the column headings. *p* values are based on Pearson’s chi-square tests unless otherwise specified. Cramér’s V is reported as the effect size for categorical comparisons. Expected-cell assumptions for the chi-square test were all satisfied for this table. The final column presents a sensitivity analysis after exclusion of the Other/unspecified disease category. For multiple-response items, participants could select more than one option; therefore, percentages may exceed 100%.

## Data Availability

The datasets presented in this article are not readily available as we did not obtain consent from the families to share this data with third parties. Consent was granted solely for the use of the data within the scope of this study.

## Supplementary Materials

The following supporting information can be downloaded at: https://www.mdpi.com/article/10.3390/jcm15176690/s1, List S1: Diagnosis-to-category mapping; Questionnaire S1: English translation of the survey; Table S1: Multivariable logistic regression analyses; Table S2: Specialist services utilized, %; Table S3: Selected subjective outcomes according to respondent type, stratified by patient age.

## Abbreviations

The following abbreviations are used in this manuscript:

NGO	Non-governmental organization
DMD	Duchenne Muscular Dystrophy
EURORDIS	European Organization for Rare Diseases
