# Supplementary material for: Patient- and Caregiver-Reported Experiences of Rare Diseases in Türkiye: A Patient-Organization-Recruited Online Survey
Source: J Clin Med. 2026 Aug 28;15(17):6690. doi: 10.3390/jcm15176690 (PMC13565767; doi:10.3390/jcm15176690)
Supplement: Supplementary file 1 [file jcm-15-06690-s001.zip › List S1.pdf]

List S1: Diagnosis-to-category mapping

1) METABOLIC AND STORAGE DISEASES

1A) Lysosomal Storage Diseases (LSD)

- Lysosomal Storage Diseases
- Gaucher disease
- Cystinosis
- Wolman disease
- Mucolipidosis
- NCL (Neuronal ceroid lipofuscinosis)

1B) Other Storage Diseases and Organelle-Related Defects

- Glycogen storage disease
- Neutral lipid storage disease

1C) Disorders of Carbohydrate Metabolism

- Galactosemia
- Fructose-1,6-bisphosphatase deficiency
- Aldolase B deficiency (Hereditary fructose intolerance)
- Fructosemia

1D) Amino Acid Metabolism, Urea Cycle Disorders, and Organic Acidemias

- Phenylketonuria (PKU)
- Tyrosinemia type I
- Alkaptonuria
- Homocystinuria
- Lysinuric protein intolerance
- MSUD (Maple syrup urine disease)
- Isovaleric acidemia
- Propionic acidemia
- Methylmalonic acidemia
- Argininemia (arginase deficiency)
- Citrullinemia
- OTC deficiency (ornithine transcarbamylase deficiency)
- Urea cycle disorder
- Albinism
- AADC deficiency (Aromatic L-amino acid decarboxylase deficiency)

1E) Congenital Disorder of Glycosylation (CDG)

1F) Porphyrias

1G) Other Metabolic Disorders

- Congenital hyperinsulinism
- COQ4 deficiency (mitochondrial; coenzyme Q biosynthesis defect)
- Smith Lemli Opitz syndrome
- POMC (proopiomelanocortin) related disorder

- Primary carnitine deficiency
- Hypophosphatemic rickets
- Lipodystrophy
- Mitochondrial membrane protein-associated neurodegeneration (MPAN)

2) NEUROMUSCULAR DISEASES

- SMA (Spinal muscular atrophy)
- DMD (Duchenne muscular dystrophy) / BMD (Becker Muscular Dystrophy)
- LGMD (Limb-girdle muscular dystrophy)
- Ullrich congenital muscular dystrophy
- Congenital myopathy / congenital muscular dystrophy (e.g., TRIP4 gene related)
- Myopathy
- Centronuclear myopathy
- Myofibrillar myopathy
- Dysferlinopathy
- Bethlem myopathy
- Facioscapulohumeral muscular dystrophy (FSHD)
- Hereditary spastic paraplegia (HSP)
- Ryanodine receptor type 2–related disorder (RYR2 related disease)
- Pelizaeus Merzbacher disease
- Cone dystrophy
- ALS (Amyotrophic lateral sclerosis)
- Myasthenia gravis

3) NEUROGENETIC AND/OR NEURODEGENERATIVE DISEASES

(Neurological–neurodevelopmental and/or neurodegenerative syndromes)

- Ataxia-telangiectasia
- Friedreich ataxia
- Spinocerebellar ataxia
- Leigh syndrome
- Rett syndrome
- Dravet syndrome
- Pitt–Hopkins syndrome
- Angelman syndrome
- FOXG1 syndrome
- KAT6A syndrome
- IQSEC2-related disorder
- SYNGAP1 gene mutation
- Pontocerebellar hypoplasia
- PRUNE1-related disorder
- Fragile X syndrome
- Huntington disease

Some syndromes have both neurodevelopmental and multisystem features; they are listed here because their dominant clinical features are neurodevelopmental.

4) CONGENITAL ANOMALIES / GENETIC-CHROMOSOMAL SYNDROMES

4A) Chromosomal Deletion/Duplication Syndromes

- Williams syndrome
- Down syndrome (Trisomy 21)
- 13q deletion syndrome
- 6q26–q27 deletion syndrome
- 4p duplication syndrome
- 5q35.2–q35.3 duplication syndrome
- Alfi syndrome (9p deletion syndrome)
- Wolf–Hirschhorn syndrome (4p deletion syndrome)

4B) Other Specific Genetic Syndromes

- Arboleda–Tham syndrome
- Beare–Stevenson cutis gyrata syndrome
- CEBALID syndrome
- CHARGE syndrome
- Cohen syndrome
- Cornelia de Lange syndrome
- DDX3X syndrome
- DiGeorge syndrome
- GNB1-related disorder
- Glass syndrome
- Gould syndrome
- Joubert syndrome
- Kallmann syndrome
- Lamb–Shaffer syndrome
- NESCAV syndrome / Delpire–McNeill syndrome
- Nicolaides–Baraitser syndrome
- Neurofibromatosis
- Osler Weber Rendu syndrome
- Shprintzen Goldberg syndrome
- Smith Magenis syndrome
- Tuberous sclerosis complex
- Van der Knaap disease
- Von Hippel Lindau disease
- Wiedemann Steiner syndrome
- Norrie syndrome
- Oculo auriculo vertebral spectrum (OAVS)
- Silver Russell syndrome
- Treacher Collins syndrome

4C) Skeletal Dysplasias / Congenital Bone Cartilage Disorders

- Achondroplasia
- Melorheostosis
- Osteopetrosis
- Stüve Wiedemann syndrome

4D) Major Congenital Anomalies

- Diaphragmatic hernia
- Bladder exstrophy
- Microtia
- Esophageal atresia
- Spina bifida

5) AUTOIMMUNE, AUTOINFLAMMATORY AND RHEUMATOLOGIC DISEASES

- Ankylosing spondylitis
- Behçet's disease
- CAPS (Cryopyrin-associated periodic syndromes)
- FMF (Familial Mediterranean fever)
- IgA nephropathy
- Neuromyelitis optica (Devic disease)
- Parry Romberg syndrome
- Psoriatic arthritis
- SAVI syndrome
- Sarcoidosis
- Sjögren syndrome
- SLE (Systemic lupus erythematosus)
- Vogt Koyanagi Harada disease

6) CARDIOVASCULAR AND/OR PULMONARY DISEASES

- Eisenmenger syndrome
- Idiopathic pulmonary fibrosis
- Cystic fibrosis
- Left ventricular noncompaction cardiomyopathy
- Post capillary pulmonary hypertension
- Primary ciliary dyskinesia
- Pulmonary hypertension
- Idiopathic interstitial pneumonia

7) OTHER / UNSPECIFIED

7a) Dermatologic and Connective Tissue Diseases

- Lichen sclerosus
- Pemphigus vulgaris
- Scleroderma
- Scleromyxedema
- Yellow nail syndrome
- Trichothiodystrophy

7b) Endocrine and Hormone Related Diseases

- Type 1 diabetes mellitus

7c) Hematologic / Immunodeficiencies

- ADA deficiency (adenosine deaminase deficiency; SCID)
- PNH (Paroxysmal nocturnal hemoglobinuria)
- Von Willebrand disease

7d) Other / Not Specifically Categorized

- Gitelman syndrome (renal tubulopathy)
- Histiocytosis
- Hereditary pancreatitis
- Pseudotumor cerebri (intracranial hypertension)
- Retinitis pigmentosa
- SSPE (Subacute sclerosing panencephalitis)

Notes on a few ambiguous eponym/acronym items (for standard English naming):

- CEBALID syndrome is also described as MN1 C-terminal truncation (MCTT) syndrome.
- Delpire–McNeill syndrome is used for an SLC12A2-related neurodevelopmental disorder (often abbreviated "DELMNES").
- "Glass syndrome" is commonly used for SATB2-associated syndrome.
- H-ABC expands to "Hypomyelination with atrophy of the basal ganglia and cerebellum."
- NESCAV syndrome is an established name (also described with neurodegeneration/spasticity ± cerebellar atrophy/cortical visual impairment).
