# Supplementary material for: Patient- and Caregiver-Reported Experiences of Rare Diseases in Türkiye: A Patient-Organization-Recruited Online Survey
Source: J Clin Med. 2026 Aug 28;15(17):6690. doi: 10.3390/jcm15176690 (PMC13565767; doi:10.3390/jcm15176690)
Supplement: Supplementary file 1 [file jcm-15-06690-s001.zip › Questionnaire S1.pdf]

Questionnaire S1. English translation of the survey

Türkiye Rare Diseases Current Situation and Needs Analysis Survey

Informed Voluntary Consent Form

Purpose of the Survey

This survey has been designed within the scope of the Turkish Ministry of Health Rare Diseases Health Strategy Document and Action Plan, under the main heading “Supporting Patients and Their Relatives and Improving Quality of Life”, objective 2.2.1: Evaluation of the direct experiences of individuals with rare diseases and their relatives regarding difficulties in diagnosis, treatment, and access to healthcare services.

The survey aims to assess the current situation and needs of individuals with rare diseases and their families in Türkiye. Approximately 1,000 participants are targeted. Your sincere responses will contribute significantly to understanding rare diseases in our country and identifying unmet needs.

The survey includes questions about the patient’s health status, treatment process, and challenges faced, and takes approximately 15–25 minutes to complete. All information provided will remain confidential and will be used solely for research purposes. No personal identifying information (such as name or email address) will be collected.

Participation is entirely voluntary, and you may withdraw at any time without consequence. For questions about the survey, please contact: shgm.nadir@saglik.gov.tr

(* indicates mandatory questions)

Please tick all that apply.

1. I declare that I have read and understood this form, that I am informed about the purpose of this research survey and how it will be conducted, that my participation is voluntary, and that I may withdraw from the study at any time if I wish.

General and Social Information

2. Please select one option only.

- The person with the rare disease
- Primary caregiver of the person with rare disease

3. Province of residence: *
4. Patient’s date of birth: *
   (Example: January 7, 2019)

5. Current status of the patient: *

- Alive
- Deceased

6. Patient's gender: *

- Female
- Male

7. Patient's education level: *

- Not of school age
- Primary school
- Secondary school
- High school
- University
- Master's / Doctorate
- Not a graduate of any educational institution

8. Monthly household income level: *

- Below 17,000 TRY
- 17,000–35,000 TRY
- 35,000–50,000 TRY
- 50,000–100,000 TRY
- Above 100,000 TRY

9. If there is a caregiver, their education level
   (If there are two or more caregivers, select the highest education level):

- No caregiver
- Primary school
- Secondary school
- High school
- University
- Master's / Doctorate
- Not a graduate of any educational institution

10. Patient's employment status:

- Not of working age
- Unable to work (due to illness or disability)
- Able to work but unemployed / unable to find a job
- Homemaker
- Student
- Public sector employee
- Private sector employee
- Retired
- Other: _______

11. Employment status of the caregiver (if any):

- No caregiver
- Unable to work (due to illness or disability)
- Able to work but unemployed / unable to find a job
- Homemaker
- Student
- Public sector employee
- Private sector employee
- Retired
- Other: _______

12. Patient's health insurance status: *

- Covered by Social Security Institution (SSI)
- Private health insurance
- SSI + private health insurance
- No health insurance

Disease Information

13. Name of the rare disease *(Diseases are listed alphabetically; if not listed, select "Other" and specify):*

- ADA (Adenosine deaminase) deficiency
- Achondroplasia
- Albinism
- ALS (Amyotrophic lateral sclerosis)
- Ataxia-Telangiectasia
- Cri-du-Chat syndrome
- DMD/BMD (Duchenne/Becker Muscular Dystrophy)
- Down syndrome
- Epidermolysis bullosa
- Phenylketonuria
- FMF (Familial Mediterranean Fever)
- Fragile X syndrome
- Huntington's disease
- Juvenile rheumatoid arthritis
- Cystic fibrosis
- Lysosomal storage diseases (e.g., mucopolysaccharidosis)
- NCL (Neuronal ceroid lipofuscinosis)
- Neurofibromatosis
- Pulmonary hypertension
- Retinitis pigmentosa
- Cystinosis
- Scleroderma
- SMA (Spinal muscular atrophy)
- Treacher Collins syndrome
- Williams syndrome
- Other: _______

14. Is there another person in your family with the same rare disease? *

- Yes, 2 people
- Yes, 3 or more people
- No

15. Had you heard of this disease before diagnosis? *

- Yes, I had heard of it
- No, I heard of it for the first time at diagnosis

16. Which sources do you use to obtain information about the disease? *(Multiple answers allowed)*

- Internet / social media
- Healthcare professionals
- Patient associations
- Other patients or relatives with the same disease
- I do not receive information from any source

17. Is the patient followed regularly at a healthcare facility? *

- Yes
- No

18. After diagnosis, did you feel the need for emotional / psychosocial / psychiatric support? *

- Yes
- No

Psychosocial Impact and Challenges

19. Have you ever received psychosocial support (psychiatrist, child psychiatrist, psychologist, social worker, etc.)? *

- Yes
- No

20. Are you a member of a disease-related association / NGO? *

- Yes, I am a member / in contact
- I do not know if such an association exists
- I searched but could not find one
- No, I am not a member

21. Which difficulties does the disease cause? *(Multiple answers allowed)*

- Daily activities (housework, cooking, shopping, etc.)
- Self-care skills (dressing, eating, personal hygiene, etc.)
- Difficulties due to loss of movement or senses (vision, hearing, posture control, etc.)
- Difficulties in school/work life
- Learning and comprehension difficulties
- Social life and relationships
- Communication with others (speaking, texting, emailing, etc.)
- Behavioral control
- I experience no difficulties

22. Problems experienced after diagnosis *(Multiple answers allowed)*

- I had to resign from my job
- My job opportunities decreased
- I had to relocate due to hospital access
- My financial situation worsened
- My social life became restricted
- My contact with relatives/friends decreased or ended
- None

23. Which aspects of rare disease were most challenging? *(Multiple answers allowed)*

- Diagnostic process
- Frequent testing
- Transportation to hospital
- Frequent hospitalizations
- Difficulty accessing specialist physicians
- Difficulty accessing medication
- Difficulty accessing home healthcare services
- Special nutrition requirements
- Difficulty communicating with patient organizations
- Not knowing what to do
- Difficulties in education or work life
- Financial difficulties
- Difficulty accessing medical devices
- Problems communicating with public institutions
- Difficulty obtaining or renewing disability reports
- I did not experience disease-related difficulties

Diagnosis Process

24. Was the disease diagnosed before birth? *

- Yes
- No

25. Was diagnosis made through a screening program?

- Yes
- No
- Not included in screening programs

26. Age at onset of symptoms: * *(Open-ended)*
    (e.g., present at birth, 1 month, 1.5 years, 12 years)
27. Age at definitive diagnosis: * *(Open-ended)*
28. Number of different physicians consulted before definitive diagnosis: *

- None (no symptoms at diagnosis)
- 1
- 1–3
- 4–10
- 11–20
- More than 20

29. Was an incorrect or different diagnosis made before definitive diagnosis? *

- Yes
- No

30. Did you seek a second opinion after initial diagnosis? *

- No
- Yes, from 1 additional physician
- Yes, from 2 additional physicians
- Yes, from more than 3 physicians

31. Do you think the diagnosis could have been made earlier? *

- Yes
- No

32. If yes, why was diagnosis delayed? *(Multiple answers allowed)*

- Difficulty accessing hospital
- Referral problems to specialists
- Lack of disease knowledge among healthcare professionals
- Diagnostic tests could not be performed
- Tests performed too late

Healthcare Services and Access

33. How adequately were you informed by healthcare professionals? *

- Not informed at all
- Very little informed
- Moderately informed

- Adequately informed
- Very well informed

34. Which areas could have been better regarding information provision? *(Multiple answers allowed)*

- No deficiencies
- Communication style
- Lack of support / counseling
- Insufficient disease information
- Too little time spent with healthcare professional

35. Additional comments on the diagnostic process (positive or negative): *(Open-ended)*
36. Number of healthcare visits in the last year: * *(Single number)*
37. Number of hospitalization days in the last year: * *(Single number)*
38. Can you meet healthcare needs related to the disease in your province? *

- Yes, in my province
- Occasionally need to travel to another province
- Mostly need to travel to another province
- Always need to travel to another province

39. Which hospital do you most frequently visit for disease-related care? *
    (Please specify province and hospital name)

Healthcare Utilization (Last 1 Year)

40. Indicate frequency of visits to the following specialties if present:
    (1–2 / 3–5 / 5–10 / >10)

- Family Medicine
- Emergency Department
- Pediatrics / Internal Medicine
- Ophthalmology
- ENT
- Dermatology
- Orthopedics
- Cardiology
- Neurology
- Psychiatry
- Metabolism
- Pulmonology
- Thoracic Surgery
- Gastroenterology
- Endocrinology
- Nephrology
- Hematology
- Rheumatology

- Allergy / Immunology
- Medical (or Pediatric) Genetics
- General Surgery / Pediatric Surgery
- Neurosurgery
- Infectious Diseases
- Radiology
- Oncology
- Algology
- Obstetrics & Gynecology
- Urology
- Physical Therapy & Rehabilitation
- Physiotherapist
- Dentist
- Speech and Language Therapist
- Occupational Therapist
- Dietitian

Treatment and Expectations

41. Do you find access to current treatment services sufficient? *

- Not sufficient at all
- Insufficient
- Moderately sufficient
- Sufficient
- Very sufficient

42. If insufficient, which areas are lacking? *(Multiple answers allowed)*

- Distance to hospital
- Another dependent person needing care at home
- Lack of transportation
- Unable to get leave from work
- Not knowing where/which department to go
- Required service not provided

43. Most important needs in diagnosis and treatment: *(Multiple answers allowed)*

- Access to accurate disease information
- Access to appropriate specialists
- Access to medications
- Access to medical devices
- Communication with similar patients/groups
- Information on new treatment trials
- Home healthcare services

44. Most important factors to improve quality of diagnosis and treatment: *(Multiple answers allowed)*

- Establishment of specialized centers
- Increasing healthcare professionals' knowledge
- Improving patient–provider communication
- Facilitating transition from pediatric to adult care
- Information and access to social services
- Faster access to newly approved drugs
- More effective use of family physicians
