# Supplementary material for: Patient- and Caregiver-Reported Experiences of Rare Diseases in Türkiye: A Patient-Organization-Recruited Online Survey
Source: J Clin Med. 2026 Aug 28;15(17):6690. doi: 10.3390/jcm15176690 (PMC13565767; doi:10.3390/jcm15176690)
Supplement: Supplementary file 1 [file jcm-15-06690-s001.zip › Table S1.pdf]

Table S1. Multivariable Logistic Regression Analyses

A) Multivariable logistic regression analyses of factors associated with perceived need for emotional, psychosocial, or psychiatric support at or after diagnosis: sensitivity analyses

**Model 1: Overall, N=1642**				
	**Sig.**	**Exp(B)**	**95% C.I.for EXP(B)**	
n=1642			**Lower**	**Upper**
Caregiver vs. self	**0,003**	**1,75**	**1,21**	**2,54**
Patient's age, yrs	0,099	0,99	0,98	1,00
Patient's sex, male vs. female	0,156	0,83	0,65	1,07
Disease categories (ref: Metabolic and Storage Dis)	0,231			
Neuromuscular Dis	0,811	1,04	0,78	1,37
Neurogenetic and/or Neurodegenerative Dis	0,732	1,07	0,71	1,61
Congenital Anomalies / Syndromes	0,016	1,83	1,12	2,98
Autoimmune, Autoinflammatory and Rheumatologic	0,864	0,95	0,53	1,70
Cardiovascular and/or Pulmonary Dis	0,172	1,52	0,83	2,77
Other / Unspecified	0,449	1,31	0,65	2,66
Constant	0,001	2,30		

**Model 2: Overall sample excluding the other/unspecified disease group, n=1597**				
	**Sig.**	**Exp(B)**	**95% C.I.for EXP(B)**	
			**Lower**	**Upper**
Caregiver vs. self	**0,002**	**1,83**	**1,25**	**2,68**
Patient's age, yrs	0,096	0,99	0,98	1,00
Patient's sex, male vs. female	0,093	0,80	0,62	1,04
Disease categories (ref: Metabolic and Storage Dis)	0,184			
Neuromuscular Dis	0,735	1,05	0,79	1,39
Neurogenetic and/or Neurodegenerative Dis	0,739	1,07	0,71	1,61
Congenital Anomalies / Syndromes	**0,018**	**1,81**	**1,11**	**2,96**
Autoimmune, Autoinflammatory and Rheumatologic	0,931	0,98	0,54	1,75
Cardiovascular and/or Pulmonary Dis	0,158	1,54	0,85	2,82
Constant	0,001	2,28		

**Model 3: Participants diagnosed after symptom onset, n=1388**				
	**Sig.**	**Exp(B)**	**95% C.I.for EXP(B)**	
			**Lower**	**Upper**
Caregiver vs. self	**0,004**	**1,77**	**1,20**	**2,61**
Patient's age, yrs	0,117	0,99	0,98	1,00
Patient's sex, male vs. female	0,040	0,75	0,57	0,99
Disease categories (ref: Metabolic and Storage Dis)	0,246			
Neuromuscular Dis	0,757	1,05	0,77	1,45
Neurogenetic and/or Neurodegenerative Dis	0,884	1,03	0,67	1,60
Congenital Anomalies / Syndromes	**0,013**	**2,14**	**1,17**	**3,90**
Autoimmune, Autoinflammatory and Rheumatologic	0,933	0,97	0,53	1,79
Cardiovascular and/or Pulmonary Dis	0,322	1,39	0,73	2,65
Other / Unspecified	0,374	1,40	0,67	2,92
Constant	0,001	2,47		

B) Multivariable logistic regression analyses of factors associated with previous receipt of psychosocial support from health professionals: sensitivity analyses

Model 1: Overall, N=1642				
	Sig.	Exp(B)	95% C.I.for EXP(B)	
			Lower	Upper
Caregiver vs. self	0,184	1,28	0,89	1,83
Patient's age, yrs	0,180	1,01	1,00	1,02
Patient's sex, male vs. female	0,428	0,91	0,73	1,14
Disease categories (ref: Metabolic and Storage Dis)	0,000			
Neuromuscular Dis	0,797	0,97	0,74	1,26
Neurogenetic and/or Neurodegenerative Dis	**0,041**	**1,46**	**1,02**	**2,09**
Congenital Anomalies / Syndromes	**0,000**	**2,07**	**1,43**	**2,99**
Autoimmune, Autoinflammatory and Rheumatologic	**0,029**	**1,85**	**1,07**	**3,23**
Cardiovascular and/or Pulmonary Dis	0,932	1,02	0,60	1,76
Other / Unspecified	0,522	1,24	0,64	2,38
Constant	0,000	0,36		

Model 2: Overall sample excluding the other/unspecified disease group, n=1597				
	Sig.	Exp(B)	95% C.I.for EXP(B)	
			Lower	Upper
Caregiver vs. self	0,128	1,33	0,92	1,93
Patient's age, yrs	0,069	1,01	1,00	1,02
Patient's sex, male vs. female	0,529	0,93	0,74	1,17
Disease categories (ref: Metabolic and Storage Dis)	0,000			
Neuromuscular Dis	0,757	0,96	0,74	1,25
Neurogenetic and/or Neurodegenerative Dis	**0,041**	**1,46**	**1,02**	**2,09**
Congenital Anomalies / Syndromes	**0,000**	**2,09**	**1,45**	**3,02**
Autoimmune, Autoinflammatory and Rheumatologic	**0,038**	**1,80**	**1,03**	**3,13**
Cardiovascular and/or Pulmonary Dis	0,960	0,99	0,57	1,70
Constant	0,000	0,33		

Model 3: Participants diagnosed after symptom onset, n=1388				
	Sig.	Exp(B)	95% C.I.for EXP(B)	
			Lower	Upper
Caregiver vs. self	0,235	1,26	0,86	1,84
Patient's age, yrs	0,466	1,00	0,99	1,02
Patient's sex, male vs. female	0,285	0,87	0,68	1,12
Disease categories (ref: Metabolic and Storage Dis)	0,001			
Neuromuscular Dis	0,542	0,91	0,68	1,23
Neurogenetic and/or Neurodegenerative Dis	0,153	1,33	0,90	1,95
Congenital Anomalies / Syndromes	**0,001**	**2,12**	**1,38**	**3,27**
Autoimmune, Autoinflammatory and Rheumatologic	0,065	1,72	0,97	3,06
Cardiovascular and/or Pulmonary Dis	0,485	0,80	0,44	1,48
Other / Unspecified	0,532	1,24	0,63	2,42
Constant	0,001	0,42		

C) Multivariable logistic regression analyses of factors associated with history of receiving an incorrect or alternative diagnosis prior to confirmation: sensitivity analyses

Model 1: Overall, N=1642				
	Sig.	Exp(B)	95% C.I.for EXP(B)	
			Lower	Upper
Caregiver vs. self	**0,040**	**0,68**	**0,48**	**0,98**
Patient's age, yrs	**0,000**	**1,03**	**1,01**	**1,04**
Patient's sex, male vs. female	0,137	1,21	0,94	1,55
Disease categories (ref: Metabolic and Storage Dis)	0,000			
Neuromuscular Dis	0,413	1,13	0,85	1,51
Neurogenetic and/or Neurodegenerative Dis	**0,000**	**2,48**	**1,70**	**3,63**
Congenital Anomalies / Syndromes	0,191	1,33	0,87	2,05
Autoimmune, Autoinflammatory and Rheumatologic	**0,000**	**6,44**	**3,38**	**12,29**
Cardiovascular and/or Pulmonary Dis	0,088	1,61	0,93	2,78
Other / Unspecified	**0,035**	**2,02**	**1,05**	**3,88**
Constant	0,000	0,24		

Model 2: Overall sample excluding the other/unspecified disease group, n=1597				
	Sig.	Exp(B)	95% C.I.for EXP(B)	
			Lower	Upper
Caregiver vs. self	0,132	0,75	0,52	1,09
Patient's age, yrs	**0,000**	**1,03**	**1,02**	**1,04**
Patient's sex, male vs. female	0,184	1,19	0,92	1,53
Disease categories (ref: Metabolic and Storage Dis)	0,000			
Neuromuscular Dis	0,391	1,14	0,85	1,52
Neurogenetic and/or Neurodegenerative Dis	**0,000**	**2,47**	**1,69**	**3,61**
Congenital Anomalies / Syndromes	0,189	1,34	0,87	2,06
Autoimmune, Autoinflammatory and Rheumatologic	**0,000**	**6,43**	**3,37**	**12,27**
Cardiovascular and/or Pulmonary Dis	0,107	1,57	0,91	2,72
Constant	0,000	0,22		

Model 3: Participants diagnosed after symptom onset, n=1388				
	Sig.	Exp(B)	95% C.I.for EXP(B)	
			Lower	Upper
Caregiver vs. self	0,069	0,71	0,49	1,03
Patient's age, yrs	**0,000**	**1,02**	**1,01**	**1,04**
Patient's sex, male vs. female	0,186	1,19	0,92	1,55
Disease categories (ref: Metabolic and Storage Dis)	0,000			
Neuromuscular Dis	**0,018**	**0,69**	**0,51**	**0,94**
Neurogenetic and/or Neurodegenerative Dis	0,054	1,47	0,99	2,18
Congenital Anomalies / Syndromes	0,945	1,02	0,64	1,62
Autoimmune, Autoinflammatory and Rheumatologic	**0,000**	**4,29**	**2,20**	**8,35**
Cardiovascular and/or Pulmonary Dis	0,640	1,15	0,64	2,07
Other / Unspecified	0,388	1,34	0,69	2,61
Constant	0,001	0,41		

Model 4: Participants diagnosed after symptom onset excluding the other/unspecified disease group, n=1344				
	**Sig.**	**Exp(B)**	**95% C.I.for EXP(B)**	
			**Lower**	**Upper**
Caregiver vs. self	0,185	0,77	0,52	1,13
Patient's age, yrs	**0,000**	**1,03**	**1,01**	**1,04**
Patient's sex, male vs. female	0,236	1,17	0,90	1,53
Disease categories (ref: Metabolic and Storage Dis)	0,000			
Neuromuscular Dis	**0,020**	**0,69**	**0,51**	**0,94**
Neurogenetic and/or Neurodegenerative Dis	0,056	1,47	0,99	2,17
Congenital Anomalies / Syndromes	0,941	1,02	0,64	1,62
Autoimmune, Autoinflammatory and Rheumatologic	**0,000**	**4,28**	**2,19**	**8,33**
Cardiovascular and/or Pulmonary Dis	0,703	1,12	0,62	2,02
Constant	0,000	0,37		

D) Multivariable logistic regression analyses of factors associated with perception that the diagnosis could have been made earlier: sensitivity analyses

Model 1: Overall, N=1642				
	**Sig.**	**Exp(B)**	**95% C.I.for EXP(B)**	
			**Lower**	**Upper**
Caregiver vs. self	0,470	1,16	0,78	1,72
Patient's age, yrs	**0,020**	**1,02**	**1,00**	**1,03**
Patient's sex, male vs. female	0,927	1,01	0,79	1,29
Disease categories (ref: Metabolic and Storage Dis)	0,007			
Neuromuscular Dis	**0,036**	**1,33**	**1,02**	**1,74**
Neurogenetic and/or Neurodegenerative Dis	**0,001**	**2,02**	**1,33**	**3,08**
Congenital Anomalies / Syndromes	**0,034**	**1,55**	**1,03**	**2,33**
Autoimmune, Autoinflammatory and Rheumatologic	**0,014**	**2,55**	**1,21**	**5,41**
Cardiovascular and/or Pulmonary Dis	0,110	1,65	0,89	3,06
Other / Unspecified	0,352	1,43	0,68	3,02
Constant	0,202	1,39		

Model 2: Overall sample excluding the other/unspecified disease group, n=1597				
	**Sig.**	**Exp(B)**	**95% C.I.for EXP(B)**	
			**Lower**	**Upper**
Caregiver vs. self	0,402	1,19	0,79	1,79
Patient's age, yrs	**0,033**	**1,01**	**1,00**	**1,03**
Patient's sex, male vs. female	0,862	0,98	0,77	1,25
Disease categories (ref: Metabolic and Storage Dis)	0,003			
Neuromuscular Dis	**0,028**	**1,35**	**1,03**	**1,76**
Neurogenetic and/or Neurodegenerative Dis	**0,001**	**2,02**	**1,33**	**3,08**
Congenital Anomalies / Syndromes	**0,038**	**1,54**	**1,02**	**2,30**
Autoimmune, Autoinflammatory and Rheumatologic	**0,012**	**2,63**	**1,24**	**5,57**
Cardiovascular and/or Pulmonary Dis	0,097	1,68	0,91	3,12
Constant	0,202	1,40		

Model 3: Participants diagnosed after symptom onset, n=1388				
	Sig.	Exp(B)	95% C.I.for EXP(B)	
			Lower	Upper
Caregiver vs. self	0,143	1,39	0,90	2,15
Patient’s age, yrs	0,055	1,01	1,00	1,03
Patient’s sex, male vs. female	0,412	0,89	0,67	1,18
Disease categories (ref: Metabolic and Storage Dis)	0,413			
Neuromuscular Dis	0,075	0,74	0,53	1,03
Neurogenetic and/or Neurodegenerative Dis	0,889	1,03	0,65	1,65
Congenital Anomalies / Syndromes	0,671	0,90	0,54	1,49
Autoimmune, Autoinflammatory and Rheumatologic	0,409	1,39	0,64	3,04
Cardiovascular and/or Pulmonary Dis	0,848	0,93	0,46	1,90
Other / Unspecified	0,739	0,87	0,39	1,95
Constant	0,002	2,58		

Model 4: Participants diagnosed after symptom onset excluding the other/unspecified disease group, n=1344				
	Sig.	Exp(B)	95% C.I.for EXP(B)	
			Lower	Upper
Caregiver vs. self	0,142	1,40	0,89	2,20
Patient’s age, yrs	0,111	1,01	1,00	1,03
Patient’s sex, male vs. female	0,271	0,85	0,63	1,14
Disease categories (ref: Metabolic and Storage Dis)	0,320			
Neuromuscular Dis	0,090	0,75	0,54	1,05
Neurogenetic and/or Neurodegenerative Dis	0,900	1,03	0,65	1,65
Congenital Anomalies / Syndromes	0,629	0,88	0,53	1,47
Autoimmune, Autoinflammatory and Rheumatologic	0,360	1,44	0,66	3,15
Cardiovascular and/or Pulmonary Dis	0,920	0,96	0,47	1,97
Constant	0,001	2,71		

E) Multivariable logistic regression analyses of factors associated with perceived adequacy of access to current treatment services for the disease (Not adequate-Inadequate vs. adequate: sensitivity analyses

Model 1: Overall, N=1642				
	Sig.	Exp(B)	95% C.I.for EXP(B)	
			Lower	Upper
Caregiver vs. self	0,511	0,89	0,63	1,26
Patient’s age, yrs	0,285	0,99	0,98	1,01
Patient’s sex, male vs. female	0,716	0,96	0,77	1,20
Disease categories (ref: Metabolic and Storage Dis)	0,000			
Neuromuscular Dis	**0,000**	**0,41**	**0,32**	**0,52**
Neurogenetic and/or Neurodegenerative Dis	**0,005**	**0,60**	**0,42**	**0,86**
Congenital Anomalies / Syndromes	0,275	0,82	0,57	1,17
Autoimmune, Autoinflammatory and Rheumatologic	0,818	1,07	0,62	1,85
Cardiovascular and/or Pulmonary Dis	0,647	1,13	0,68	1,88
Other / Unspecified	0,579	0,84	0,44	1,57
Constant	0,181	1,36		

Model 2: Overall sample excluding the other/unspecified disease group, n=1597				
	**Sig.**	**Exp(B)**	**95% C.I.for EXP(B)**	
			**Lower**	**Upper**
Caregiver vs. self	0,876	0,97	0,68	1,39
Patient's age, yrs	0,356	1,00	0,98	1,01
Patient's sex, male vs. female	0,742	0,96	0,77	1,20
Disease categories (ref: Metabolic and Storage Dis)	0,000			
Neuromuscular Dis	**0,000**	**0,41**	**0,32**	**0,53**
Neurogenetic and/or Neurodegenerative Dis	**0,005**	**0,60**	**0,42**	**0,86**
Congenital Anomalies / Syndromes	0,262	0,81	0,57	1,17
Autoimmune, Autoinflammatory and Rheumatologic	0,716	1,11	0,64	1,92
Cardiovascular and/or Pulmonary Dis	0,607	1,14	0,69	1,91
Constant	0,346	1,25		
