# Supplementary material for: Patient- and Caregiver-Reported Experiences of Rare Diseases in Türkiye: A Patient-Organization-Recruited Online Survey
Source: J Clin Med. 2026 Aug 28;15(17):6690. doi: 10.3390/jcm15176690 (PMC13565767; doi:10.3390/jcm15176690)
Supplement: Supplementary file 1 [file jcm-15-06690-s001.zip › Table S2.pdf]

Table S2. Specialist services utilized, %

	Metabolic and Storage Diseases	Neuromuscular Diseases	Neurogenetic and/or Neurodegenerative Diseases	Congenital Anomalies / Syndromes	Autoimmune, Autoinflammatory and Rheumatologic Diseases	Cardiovascular and/or Pulmonary Diseases	Other / Unspecified	Total	p value
**Presence of visits to specialist / health professionals in the last year**									
Family Medicine Specialist	68.0a.b.c.d	64.0c.d	55.3b.d	78.5a	73.4a.b.c.d	66.2a.b.c.d	62.2a.b.c.d	66.1	**0.001**
Emergency Medicine Specialist	52.0a.b	46.0b	50.6a.b	65.8a	65.6a.b	62.2a.b	46.7a.b	51.7	**<0.001**
Pediatrician / Internal Medicine Specialist	24.1a	23.9a	26.5a	46.8b	31.3a.b	24.3a	31.1a.b	26.9	**<0.001**
Ophthalmologist	48.8a.b	22.5c	41.2b	62.7a.d	48.4a.b.d	43.2a.b	73.3d	39.5	**<0.001**
Otorhinolaryngologist (ENT Specialist)	33.3a.b	25.9b	29.4a.b	51.3c	31.3a.b.c	47.3a.c	31.1a.b.c	32.2	**<0.001**
Dermatologist	15.3a.b	9.0c	12.4b.c	18.4a.b	29.7a.d	20.3a.b.c.d	42.2d	14.4	**<0.001**
Orthopedic Specialist / Orthopedic Surgeon	23.1a	25.7a	27.6a	33.5a	28.1a	23.0a	28.9a	25.9	0.250
Cardiology Specialist	36.1a	49.9b	22.9c	70.3d	42.2a.b.c	71.6d	28.9a.b.c	45.1	**<0.001**
Neurology Specialist	30.0a	69.4b	70.0b	46.8c	26.6a.c	13.5a	20.0a	50.0	**<0.001**
Psychiatry Specialist	19.4a	20.7a	21.2a	38.6b	25.0a.b	21.6a.b	22.2a.b	22.4	**<0.001**
Metabolism Specialist	69.6a	8.4b	15.3b.c	18.4c	10.9b.c	8.1b.c	11.1b.c	28.5	**<0.001**
Pulmonology Specialist	17.3a	31.0b	14.7a	22.2a.b	31.3a.b	60.8c	22.2a.b	25.5	**<0.001**
Thoracic Surgery Specialist	5.3a.b	6.4a.b	1.2b	8.2a	7.8a.b	8.1a.b	2.2a.b	5.7	0.078
Gastroenterology Specialist	13.5a	11.7a	12.4a	27.8b	25.0a.b	29.7b	8.9a.b	15.1	**<0.001**
Endocrinology Specialist	25.3a	13.7b.c	9.4c	49.4d	23.4a.b.c	24.3a.b	13.3a.b.c	21.0	**<0.001**
Nephrology Specialist	19.8a	7.5b	6.5b	30.4a	21.9a	2.7b	11.1a.b	13.7	**<0.001**
Hematology Specialist	8.0a.b	4.5b	12.9a	12.0a	7.8a.b	8.1a.b	15.6a	7.7	**0.001**
Rheumatology Specialist	3.7a	3.6a	1.8a	6.3a	84.4b	23.0c	28.9c	8.4	**<0.001**
Allergy and Immunology Specialist	12.2a.b	7.0b	41.2c	17.1a	9.4a.b	17.6a	6.7a.b	13.6	**<0.001**
Medical Geneticist / Pediatric Geneticist	18.2a	15.4a	18.8a	36.7b	6.3a	16.2a	6.7a	18.1	**<0.001**
General Surgeon / Pediatric Surgeon	12.4a	10.6a	11.8a	25.9b	14.1a.b	9.5a.b	4.4a	12.7	**<0.001**
Neurosurgeon	9.0a.b	4.2c	4.7b.c	15.2a	9.4a.b.c	2.7a.b.c	4.4a.b.c	6.9	**<0.001**
Infectious Disease Specialist	9.6a.b	8.3b	11.8a.b	10.1a.b	21.9a	14.9a.b	13.3a.b	10.2	**0.020**
Radiology Specialist	11.4a.b.c.d	8.9c.d	6.5b.d	17.7a	21.9a	17.6a.b.c.d	24.4a	11.6	**<0.001**
Oncology Specialist	2.9a	2.8a	5.3a	5.7a	7.8a	2.7a	8.9a	3.7	0.062
Algology Specialist (Pain Medicine)	2.7a.b	2.5a.b	0.0b	5.1a.b	9.4a	0.0a.b	2.2a.b	2.7	**0.002**
Obstetrics and Gynecology Specialist	6.5a.b	4.5b	1.8b	7.0a.b.c	34.4d	14.9a.c.d	20.0c.d	7.1	**<0.001**
Urology Specialist	4.5a.b	4.8a.b	5.3a.b.c.d	11.4c.d	12.5b.d	0.0a.c	8.9a.b.c.d	5.6	**0.001**
Physical Medicine and Rehabilitation Specialist	19.0a	45.2b	37.6b.c	37.3b.c	31.3a.b.c	18.9a.c	20.0a.c	33.4	**<0.001**
Physiotherapist	11.4a	34.9b	30.6b.c	37.3b	12.5a.c	12.2a	11.1a.c	25.2	**<0.001**
Dentist	22.4a.b	19.7b	24.7a.b	31.6a	35.9a	21.6a.b	28.9a.b	23.1	**0.006**
Speech and Language Therapist	6.5a	9.2a.b	14.7b	29.1c	4.7a.b	2.7a.b	2.2a.b	10.2	**<0.001**
Occupational Therapist (Ergotherapist)	4.7a	5.3a	12.4b.c	24.7c	6.3a.b	0.0a	2.2a.b	7.4	**<0.001**
Dietitian	27.8a	10.3b.c	7.1c	10.8b.c	10.9a.b.c	21.6a.b	11.1a.b.c	15.8	**<0.001**

[abcd]Values in the same row with different superscript letters differ significantly from each other at the .05 level.
