# Supplementary material for: Patient- and Caregiver-Reported Experiences of Rare Diseases in Türkiye: A Patient-Organization-Recruited Online Survey
Source: J Clin Med. 2026 Aug 28;15(17):6690. doi: 10.3390/jcm15176690 (PMC13565767; doi:10.3390/jcm15176690)
Supplement: Supplementary file 1 [file jcm-15-06690-s001.zip › Table S3.pdf]

Table S3. Selected subjective outcomes according to respondent type, stratified by patient age

Outcome	<18 Patient n (%)	<18 Relative / caregiver n (%)	p	≥18 Patient n (%)	≥18 Relative / caregiver n (%)	p	Total Patient n (%)	Total Relative / caregiver n (%)	p
Need for psychosocial support	14 (93.3)	846 (78.6)	0.216†	224 (60.9)	135 (73.8)	**0.003**	238 (62.1)	981 (77.9)	**<0.001**
Received psychosocial support	10 (66.7)	363 (33.7)	**0.008**	120 (32.6)	74 (40.4)	0.070	130 (33.9)	437 (34.7)	0.782
Difficulties in school/work life	9 (60.0)	556 (51.7)	0.522	173 (47.0)	98 (53.6)	0.148	182 (47.5)	654 (51.9)	0.129
Learning/comprehension difficulties	7 (46.7)	342 (31.8)	0.220	30 (8.2)	48 (26.2)	**<0.001**	37 (9.7)	390 (31.0)	**<0.001**
Limitations in social life/relationships	7 (46.7)	585 (54.4)	0.552	196 (53.3)	107 (58.5)	0.247	203 (53.0)	692 (55.0)	0.500
Difficulties communicating with others	5 (33.3)	247 (23.0)	0.344	34 (9.2)	42 (23.0)	**<0.001**	39 (10.2)	289 (23.0)	**<0.001**
Behavioral regulation difficulties	5 (33.3)	215 (20.0)	0.201	29 (7.9)	33 (18.0)	**<0.001**	34 (8.9)	248 (19.7)	**<0.001**
Resignation from employment	3 (20.0)	142 (13.2)	0.441	30 (8.2)	25 (13.7)	**0.042**	33 (8.6)	167 (13.3)	**0.015**
Reduced employment opportunities	2 (13.3)	155 (14.4)	0.907	105 (28.5)	35 (19.1)	**0.017**	107 (27.9)	190 (15.1)	**<0.001**
Relocation due to hospital access	1 (6.7)	177 (16.4)	0.309	49 (13.3)	31 (16.9)	0.255	50 (13.1)	208 (16.5)	0.103
Deterioration in financial situation	4 (26.7)	452 (42.0)	0.232	90 (24.5)	76 (41.5)	**<0.001**	94 (24.5)	528 (41.9)	**<0.001**
Restricted social life	8 (53.3)	679 (63.1)	0.436	241 (65.5)	136 (74.3)	**0.036**	249 (65.0)	815 (64.7)	0.920
Reduced/lost contact with relatives/friends	10 (66.7)	560 (52.0)	0.260	177 (48.1)	108 (59.0)	0.016	187 (48.8)	668 (53.1)	0.147
Perceived lack of disease knowledge of healthcare professionals	7 (46.7)	503 (46.7)	0.995	209 (56.8)	101 (55.2)	0.721	216 (56.4)	604 (48.0)	**0.004**
No perceived deficiencies in information provision	2 (13.3)	192 (17.8)	0.650	60 (16.3)	33 (18.0)	0.610	62 (16.2)	225 (17.9)	0.448
Communication style identified as deficient	9 (60.0)	296 (27.5)	**0.005**	100 (27.2)	67 (36.6)	**0.023**	109 (28.5)	363 (28.8)	0.888
Lack of support/counseling	10 (66.7)	493 (45.8)	0.108	171 (46.5)	102 (55.7)	**0.040**	181 (47.3)	595 (47.3)	1.000
Insufficient disease information	8 (53.3)	583 (54.2)	0.948	215 (58.4)	106 (57.9)	0.911	223 (58.2)	689 (54.7)	0.228
Insufficient time with healthcare professional	10 (66.7)	492 (45.7)	0.106	173 (47.0)	105 (57.4)	**0.022**	183 (47.8)	597 (47.4)	0.901
Inability to obtain leave from work	2 (13.3)	189 (17.6)	0.668	43 (11.7)	32 (17.5)	0.061	45 (11.7)	221 (17.6)	0.007
Access to accurate disease information	11 (73.3)	827 (76.9)	0.748	282 (76.6)	146 (79.8)	0.403	293 (76.5)	973 (77.3)	0.750
Access to appropriate specialists/health professionals	9 (60.0)	818 (76.0)	0.150	284 (77.2)	130 (71.0)	0.117	293 (76.5)	948 (75.3)	0.631
Access to medications	9 (60.0)	701 (65.1)	0.678	265 (72.0)	137 (74.9)	0.478	274 (71.5)	838 (66.6)	0.068
Access to medical devices	4 (26.7)	453 (42.1)	0.229	136 (37.0)	82 (44.8)	0.076	140 (36.6)	535 (42.5)	0.039
Communication with similar patients/groups	9 (60.0)	560 (52.0)	0.540	170 (46.2)	89 (48.6)	0.589	179 (46.7)	649 (51.5)	0.099
Information about new treatment trials	8 (53.3)	783 (72.8)	0.094	264 (71.7)	146 (79.8)	**0.042**	272 (71.0)	929 (73.8)	0.284
Utilization of home healthcare services	4 (26.7)	336 (31.2)	0.705	109 (29.6)	68 (37.2)	0.074	113 (29.5)	404 (32.1)	0.340
Establishment of specialized rare-disease centers	12 (80.0)	867 (80.6)	0.955	281 (76.4)	145 (79.2)	0.448	293 (76.5)	1012 (80.4)	0.100
Increasing healthcare professionals' disease knowledge	11 (73.3)	888 (82.5)	0.353	313 (85.1)	149 (81.4)	0.275	324 (84.6)	1037 (82.4)	0.311

Improving patient–healthcare professional communication	8 (53.3)	702 (65.2)	0.337	234 (63.6)	121 (66.1)	0.559	242 (63.2)	823 (65.4)	0.433
Facilitating transition from pediatric to adult care	7 (46.7)	388 (36.1)	0.396	111 (30.2)	83 (45.4)	**<0.001**	118 (30.8)	471 (37.4)	**0.018**
Information/access to available social services	7 (46.7)	669 (62.2)	0.219	222 (60.3)	107 (58.5)	0.676	229 (59.8)	776 (61.6)	0.516
Faster bureaucratic access to newly available medications	10 (66.7)	774 (71.9)	0.652	293 (79.6)	145 (79.2)	0.916	303 (79.1)	919 (73.0)	**0.016**
More effective use of family physician services	8 (53.3)	380 (35.3)	0.148	138 (37.5)	69 (37.7)	0.963	146 (38.1)	449 (35.7)	0.381

Note: Data are n (% within respondent type). Pearson's χ^2 test was used unless expected cell frequencies required Fisher's exact test. †Fisher's exact test.
